# An alternative angiosperm DGAT1 topology and potential motifs in the N-terminus

**DOI:** 10.3389/fpls.2022.951389

**Published:** 2022-09-16

**Authors:** Somrutai Winichayakul, Amy Curran, Roger Moraga, Ruth Cookson, Hong Xue, Tracey Crowther, Marissa Roldan, Greg Bryan, Nick Roberts

**Affiliations:** ^1^Resilient Agriculture Innovation Centre of Excellence, AgResearch Ltd., Palmerston North, New Zealand; ^2^ZeaKal Inc., San Diego, CA, United States; ^3^Bioinformatics and Statistics, AgResearch Ltd., Palmerston North, New Zealand

**Keywords:** diacylglycerol acyltransferase, DGAT1, topology, seed oil accumulation, N-terminal motifs

## Abstract

The highly variable cytoplasmic N-terminus of the plant diacylglycerol acyltransferase 1 (DGAT1) has been shown to have roles in oligomerization as well as allostery; however, the biological significance of the variation within this region is not understood. Comparing the coding sequences over the variable N-termini revealed the Poaceae DGAT1s contain relatively high GC compositional gradients as well as numerous direct and inverted repeats in this region. Using a variety of reciprocal chimeric DGAT1s from angiosperms we show that related N-termini had similar effects (positive or negative) on the accumulation of the recombinant protein in *Saccharomyces cerevisiae*. When expressed in *Camelina sativa* seeds the recombinant proteins of specific chimeras elevated total lipid content of the seeds as well as increased seed size. In addition, we combine N- and C-terminal as well as internal tags with high pH membrane reformation, protease protection and differential permeabilization. This led us to conclude the C-terminus is in the ER lumen; this contradicts earlier reports of the cytoplasmic location of plant DGAT1 C-termini.

## Introduction

Human nutrition and oleochemical industry requirements are driving efforts to sustainably elevate the biological production of triacylglycerol (TAG) – one of the most energy dense forms of renewable reduced carbon. Pivotal to the success of this endeavor is our understanding of the endogenous mechanisms regulating TAG formation, storage and degradation.

Eukaryotes use TAG for a variety of purposes including short and long-term energy storage; lipid detoxification; and as a source of fatty acids for membrane biosynthesis (Sandager et al., 2002; Weselake, 2005; Turkish and Sturley, 2007; Chapman et al., 2013). The last step of TAG biosynthesis is an important determinant of carbon flux into TAG (Liu et al., 2012) and can be achieved by acyl-CoA-dependent or acyl-CoA-independent processes. The former uses acyl-CoA:diacylglycerol acyltransferase (DGAT; EC 2.3.1.20) while phospholipid:diacylglycerol acyltransferase (PDAT) and diacylglycerol:diacylglycerol transacylase (DGTA) perform the latter. PDAT is common to both plants and yeast (Dahlqvist et al., 2000; Ståhl et al., 2004) and some PDATs have been reported to also possess a low level of DGTA activity (Ståhl et al., 2004; Ghosal et al., 2007; Yoon et al., 2012). Specific DGTA activity, however, has only been found in animals and plants (Lehner and Kuksis, 1995; Stobart et al., 1997) and no clones have been reported to date. Of the three TAG synthesizing enzymes only the DGATs are common to animals, plants and yeast; and only DGAT1 and DGAT2 are found in all three kingdoms.

Diacylglycerol acyltransferase 1 belongs to a family of membrane-bound O-acyltransferases and was originally cloned from mouse (Cases et al., 1998) and subsequently from Arabidopsis (Hobbs et al., 1999). For the next decade it appeared that DGAT1 was not present in yeast; however, it was eventually reported in the oleaginous species *Yarrowia lipolytica* (Beopoulos et al., 2012; Zhang et al., 2012). DGAT2 has no homology to DGAT1 and is thought to have evolved separately (Turchetto-Zolet et al., 2011). DGAT2 belongs to a family that includes monoacylglycerol acyltransferases and wax ester synthases and was initially isolated from mouse (Cases et al., 2001), *Caenorhabditis elegans*, Arabidopsis and the oleaginous fungus *Mortierella ramanniana* (Lardizabal et al., 2001).

Today, functional orthologs of both DGAT1 and DGAT2 have been isolated from many organisms and their roles appear to be relatively diverse. Determining their individual contribution to TAG production in the seeds of some species is difficult due to compensation from one in the absence of the other (Routaboul et al., 1999; Zou et al., 1999); similarly, when determining the contribution of PDAT (Bates and Browse, 2012; Chapman and Ohlrogge, 2012; Banaś et al., 2013). In many oil seed species however, DGAT2 appears to be predominantly involved in the synthesis of unique TAG species whereas DGAT1 is responsible for bulk TAG biosynthesis (Cahoon et al., 2007; Liu et al., 2012). In comparison, the mammalian DGAT2 is specialized for the esterification of nascent diglycerides generated from the glycerol-3-phosphate pathway and *de novo* synthesized fatty acids, whereas both DGAT1 and DGAT2 can esterify long-chain fatty acids to diglycerides generated through hydrolysis of TAG (Qi et al., 2012; Wurie et al., 2012; Zammit, 2013; Irshad et al., 2017).

In our laboratory we are not only interested in increasing TAG accumulation in seeds we also seek to manipulate plants to accumulate TAG in vegetative tissues (Winichayakul et al., 2008, 2013, 2020; Scott et al., 2010; Beechey-Gradwell et al., 2020, 2022; Cooney et al., 2021). Of the enzymes responsible for TAG production, we are particularly interested in DGAT1 since it has a role in vegetative tissues (Kaup et al., 2002; Lu et al., 2003; Lung and Weselake, 2006) and is amenable to mutational improvements (Xu et al., 2008; Zheng et al., 2008; Meyer et al., 2009; Siloto et al., 2009; Liu et al., 2012; Greer et al., 2015; Roesler et al., 2016; Chen et al., 2017). In addition, plant DGAT1s typically have a broad substrate specificity and can enhance seed oil content and seed weight when over-expressed in the seed (Jako et al., 2001; Weslelake et al., 2007; Xu et al., 2008; Taylor et al., 2009). The results, however, have varied widely and appear to be influenced by numerous factors including: the cDNA utilized; the species transformed; and the growth conditions considered (Weselake et al., 2009). The DGAT1s from the monocotyledonous species group into a separate clade from the dicotyledonous DGAT1s (Turchetto-Zolet et al., 2011; Bhunia et al., 2021). In important monocotyledonous crop species DGAT1 has been shown to be an essential determinant of the oil levels, e.g., in maize, the domestication or breeding selected away from a high-oil QTL encoded by a DGAT1-2 allele with a phenylalanine insertion at position 469 (F469) (Zheng et al., 2008). It was subsequently demonstrated that the oil content of modern inbred maize lines could be elevated by marker assisted backcrossing to re-introduce the high-oil DGAT1-2 allele (Chai et al., 2012).

While the structure of DGAT1 is not known the hydrophilic N-terminus of both angiosperm and mammalian DGAT1s have an intrinsically disordered region (IDR) as well as a folded portion (Caldo et al., 2017). The IDR was shown to be involved in oligomerization and autoinhibition (Weselake et al., 2006; Caldo et al., 2017), whereas the folded portion is involved in positive cooperativity with acyl-CoA which may modulate allostery (Siloto et al., 2008, 2010; Caldo et al., 2017). The IDR of the angiosperm DGAT1s has a high sequence variability while the same region in the mammalian DGAT1s is relatively conserved (Lung and Weselake, 2006). The functional significance of the variation in the N-terminus of angiosperm DGAT1s is unknown. In rice however, the intrinsically disordered region of the N-terminus was thought to have a regulatory role; potentially through phosphorylation (Bhunia et al., 2021). Given our interest in the agronomic manipulation of TAG we set out to investigate this region by synthesizing C-terminally tagged, optimized versions of DGAT1 originally from *Arabidopsis thaliana*; *Tropaeolum majus*; *Oryza sativa*; and *Zea mays*. The constructs were further modified to generate reciprocal chimeras as well as N-terminally truncated (ΔN) forms and a series containing an additional N-terminal and a uniquely located internal tag. These were expressed in a variety of heterologous expression systems (*Saccharomyces cerevisiae*, *Lolium perenne*, and *Camelina sativa*) to investigate the topology, fatty acid production, recombinant protein accumulation, quaternary structure and subcellular location of DGAT1.

## Materials and methods

### Sequence searches

The majority of sequences were found using BLAST (Altschul et al., 1990) on the NCBI web site^1^ (Supplementary Table 1). However, the *Azolla filiculoides* sequence Azfi_s0008.g011645 was found at www.fernbase.org searching the *Azolla filiculoides* transcripts v1.1 while the *Pinus taeda* sequence (scaffold 892578.2) was found using http://congenie.org/blast.

### Phylogenetic analysis of exon 1 peptide sequences from plant diacylglycerol acyltransferase 1’s

Sequences were aligned by Geneious version 8.1.5 (^2^Kearse et al., 2012) Global alignment with free end gaps and Blosum62 as the Cost Matrix. An unrooted phylogenetic tree was then built using Neighbor-Joining and Jukes-Cantor as the Genetic Distance Model.

### Analysis of Poaceae N-terminal diacylglycerol acyltransferase 1 GC gradient regions

Sequences of the palindromic repeats within the Poaceae DGAT1 5′ GC gradient regions were used to search the NCBI non-redundant database (Pruitt et al., 2007) for sequences in the monocot database identical or differing from the provided query sequence by a single base. This was done in order to better represent the variability found in the same palindromic repeats anywhere else in the plant genomes; thereby increasing the sensitivity of the subsequent searches. All the selected sequences were aligned to create a Hidden Markov Model Profile with the HMMER 2.3.2 package (Eddy, 1998), constructed with a biased exit-penalty to correctly identify uneven palindromes in the DNA sequence.

### Construction of diacylglycerol acyltransferase 1 expression vectors for yeast

All DGAT1 coding sequences contained an in-frame C-terminus V5 epitope linked to a 6× histidine (6×His) tag. These were optimized for expression in yeast in order to circumvent any transcriptional and translational control which might have been present in the native sequences and synthesized by GENEART. To facilitate expression cassette construction without altering the translated sequence, in each DGAT1 we engineered an *Eco*RI site into the 5′ UTR, an *Xho*I site as part of the coding sequence for the conserved residues Leu Ser Ser in the middle of the acyl-CoA binding domain and a *Xba*I site after the stop codon.

The DNA of the yeast optimized *Arabidopsis thaliana* DGAT1 (*AtDGAT1*) was amplified by PCR, using the forward primer: 5′-GCCGCATTTAATTAAGAATTC-3′ and reverse primer: 5′-GGCAGACATAGAACCCTTTCTA-3′ and subsequently cloned into the pYES2.1/V5-His-TOPO yeast expression vector (Life Technologies, K4150-01) as per the manufacturer’s instructions. This generated AtDGAT1 with an in-frame V5:6×His C-terminal tail. The orientation of the insert and integrity of the amplicon were confirmed by restriction mapping and DNA sequencing. The remaining full length DGAT1 parents were subsequently cloned into pYES2.1 by exchanging *Eco*RI/*Xba*I digested fragment in the pYES2.1 vector containing the AtDGAT1:V5:6×His cassette. Chimeric DGAT1s were generated by exchanging the *Xho*I and *Xba*I digested fragments between the different parent constructs. The N-terminal truncated DGAT1s were created by replacing the *Eco*RI/*Xho*I fragment from each full length DGAT1 with a fragment encoding for MGGGS followed by the relevant 19 residues upstream of the conserved LSS sequence in the acyl-CoA binding domain.

Ten constructs of ZmL DGAT1 containing an N-terminal X-press epitope and a C-terminal V5 epitope were designed and optimized for expression in *S. cerevisiae*; nine of these contain an additional uniquely located internal HA epitope (indicated by the position of the HA epitopes). All DGAT1s expressed in yeast were under the control of the inducible *Gal1* promoter. The full length, chimeric, ΔN DGAT1, and internally HA tagged peptide sequences expressed in yeast are listed in Supplementary Table 1.

### Yeast strain, growth conditions and transformation

*Saccharomyces cerevisiae* quadruple mutant strain H1246 (Sandager et al., 2002) was grown at 28°C in yeast extract-peptone-dextrose medium with 250 rpm shaking. This strain cannot produce its own neutral lipids, thus any TAG that accumulates can be attributed to the recombinant protein. For growth on plates 2% (w/v) agar was added to the media. Expression vectors were transformed into the yeast cells using the S.c. EasyComp™ transformation kit (Life Technologies, K5050-01). Transformants were selected and cultured in an uracil dropout synthetic minimum medium (UdMM) containing raffinose. For DGAT1 gene induction, the transformed cells were diluted to optical density (OD_600_) of 0.4 in the UdMM containing 1% raffinose and 2% galactose. For each DGAT1 the influence on growth (cell dry weight per liter, DW/L) and total FA as a percentage of DW was measured 8, 24, and 48 h after induction; DGAT1 activity and TAG quantity were determined at 24 and 48 h respectively. Total cell proteins were analyzed by SDS-PAGE immunoblot on samples taken at 8, 24, and 48 h after induction; a similar analysis was performed on microsomal proteins from the 24 and 48h samples.

### Yeast crude cell extract for protein analysis

Total crude extract was prepared by disrupting the cells in 0.1 M NaOH and 1% (w/v) SDS as described by Kushnirov (2000). Protein samples were prepared by mixing the total crude cell extract with an equal volume of 2× loading buffer (2×LB) (Winichayakul et al., 2013), and subjected to a temperature of 37°C for 20 min. Proteins were separated by SDS-PAGE (Bio-Rad, 4–15% Mini-PROTEAN^®^ TGX stain-free™ gel) and analyzed by immunoblotting.

### Microsome protein preparation

Yeast cells expressing DGAT1 proteins or vector control were collected by centrifugation and the pellet was washed once with ice-cold H_2_O containing 1 mM phenylmethylsulfonyl fluoride (PMSF). Microsome protein was prepared as described by Ro et al. (2002). The microsome pellet was washed twice with ice-cold phosphate buffered saline (PBS) or ice-cold reaction buffer (RB, 20 mM Tris-HCl pH 7.6, 250 mM sucrose) and subjected for further protease protection assay or *in vitro* DGAT1 activity assay.

To enrich the reformed membrane sheets and provide access to luminal domains, the intact membrane compartments were agitated at 1 mg/ml in 200 mM Na_2_CO_3_, pH 11, with five passes through an insulin syringe as described by Wu et al. (2003). The resuspended pellet was incubated on ice for 1 h. The unsealed membrane sample was collected, washed twice with ice-cold PBS and subjected for protease protection assay. Microsomal protein was quantified using Qubit^®^ protein assay kit system (Life technologies, Q33211).

### Immunoblot analyses

Protein samples were separated by SDS-PAGE on 4–15% gradient polyacrylamide gels and transferred to PVDF membranes (Bio-Rad Trans-blot Turbo system). Immunoblotting was performed as described in Winichayakul et al. (2013). PVDF membranes were incubated with antibodies using the following manufacturers and dilutions: mouse anti-V5 (Life Technologies, R96025), 1:10000; rabbit anti-Kar2 (Santa Cruz Biotech, sc33630), 1:2500; anti-mouse IgG-HRP (Life Technologies, 626520), 1:5000; anti-rabbit IgG-HRP (Sigma, A6154), 1:5000; Mouse anti-Xpress (Thermo Fisher Scientific, R90125), 1:5000; mouse anti-HA (Sigma-Aldrich H3663), 1:5000; rabbit anti-BiP (Sapphire Bioscience 125-09481), 1:5000. Protein-antibody complexes were visualized using the Advansta Western Bright ECL spray (K12049-D50) and ChemiDoc MP Imager (Bio-Rad).

### *In vitro* cross-linking

Microsomal protein (1 μg/μL) in PBS was incubated with disuccinimidyl suberate (DSS, Thermo Scientific Pierce) at a final concentration of 1 mM for 50 min at 21°C. DSS was formulated at 2.5% in DMSO. Reactions were terminated by the addition of 1/10 volume of 1 M glycine (pH 7.5). An equal volume of 2×LB was added and samples were then separated by SDS-PAGE (Bio-Rad, 4–15% Mini-PROTEAN^®^ TGX stain-free™ gel) and analyzed by immunoblotting.

### Protease protection assay in *S. cerevisiae*

Fifty μg of microsomal protein prepared from each *S. cerevisiae* culture were subjected to protease protection assays. The reactions were made up to the final volume of 40 μL with 1×PBS. Reactions contained either trypsin or proteinase K (PNK, at the indicated concentration), and/or 1% Triton X-100 (TX100), and/or 50 μg/mL of digitonin (DIG) and incubated at 30°C for 30 min for trypsin digestion, or 37°C for 30 min for PNK digestion. At the end of reaction, 10 μL of 2 mg/mL trypsin inhibitors or 2 μL of 100 mM PMSF was added as a protease inhibitor, and 50 μL of 2×LB were added to the samples, and incubated at 37°C for 20 min. Samples were subjected to SDS-PAGE analysis and immunoblotting. Kar2 served as the marker protein for the yeast ER lumen (Rose et al., 1989).

### Differential permeabilization in *S. cerevisiae* and *L. perenne*

Cells expressing the vector only (VC) or tagged recombinant DGAT1 were permeabilized with either DIG or TX100 then probed with antibodies raised against either V5, Kar2 or HA, followed by the appropriate secondary antibody.

Freshly grown yeast cells of 24 h culture were grown to an OD_600_ of 4 and harvested by centrifugation at 100 × *g* at room temperature for 1 min. Cells were washed once with 0.2 mL of sterile water containing 1 mM PMSF and fixed by adding 0.18 mL of 3.5% (w/v) formaldehyde in PBS, followed by 20 μL of 1 M potassium phosphate buffer pH 6.5. After 90 min at 25°C, the fixed cells were harvested by centrifugation at 100 × *g* at RT for 1 min and the fixatives were removed by washing three times with 0.1 M potassium phosphate pH 6.5. The walls of the fixed cells were digested for 90 min at 30°C in 0.2 mL of 10% sorbitol in PBS containing 2000 Units/mL of lyticase. Fifty μL of spheroplasts were applied to polylysine coated slides and fixed by dropping 50 μL of −20°C cooled acetone. Fixed spheroplasts were immediately washed once with 0.4 mL of PBS. Cell plasma membranes were selectively permeabilized with 0.2 mL of 50 μg/mL DIG in PBS for 10 min on ice. Alternatively, total cellular membranes were fully permeabilized with 1% TX100 for 10 min at RT.

Leaf material (approximately 5 mm^2^) of transgenic *L. perenne* expressing *Tropaeolum majus* DGAT1 (Beechey-Gradwell et al., 2020; Winichayakul et al., 2020) was harvested and fixed with 3.5% paraformaldehyde in PBS using vacuum for 10 min and stored at 4°C for overnight. Fixed leaves were washed 3 times with PBS, 5 min each at RT to remove the fixative. The fixed leaf was selectively permeabilized with 0.5 mL of PBS containing 50 μg/mL DIG for 30 min on ice. Alternatively, total cellular membranes were permeabilized with 1% TX100/PBS for 30 min at RT under gentle shaking.

After permeabilization, both yeast spheroplasts and ryegrass leaf were washed 3 times with 0.5 mL of PBS and incubated with 1% (w/v) bovine serum albumin (BSA) in PBS for 10 min to block non-specific antibody binding. The spheroplasts and leaf were incubated at room temperature for 1 h with 0.2 mL of the appropriate primary antibody diluted 1/200 in BSA/PBS and washed five times with PBS. Fluorescently conjugated secondary antibodies [anti-rabbit-FITC (Life Technologies, F2765), anti-mouse-FITC (Life Technologies, 616511), anti-mouse-Cy3 (Life Technologies, A10521)] were added to the spheroplasts and leaf at 1/1000 dilution in BSA/PBS, and incubated at RT for 1 h, then quickly washed three times with PBS, further washed twice for 5 min each, and quickly rinsed in two times PBS. SlowFade gold antifade reagent with 4′,6-diamidino-2-phenylindole (DAPI, Life Technologies, S36938) was added to protect the photo-bleaching and to stain nuclei before a cover slip was added. Immunofluorescence was visualized by confocal microscopy with the excitation/emission max (Ex/Em) set at 359/461 nm for DAPI, and Ex/Em set at 495/519 nm for FITC, and Ex/Em set at 547/567 nm for Cy3 fluorescence.

### Diacylglycerol acyltransferase 1 specific activity assay

Fifty μg of microsomal protein was made up to 50 μL in RB, 50 μL of 2×LB was added and the sample heated at 37°C for 20 min. Proteins were separated by SDS-PAGE (Bio-Rad), and the quantity of recombinant DGAT1 was determined by scanning the immunoblots with a ChemiDoc MP Imaging System (Bio-Rad) and quantifying with Image Lab Software, version 4.1 (Bio-Rad). The specific activity was modified from McFie and Stone (2011) and used a fluorescent 16-[(7-nitro-2-1,3-benzoxadiazol-4-yl) amino] labeled hexadecanoyl Coenzyme A substrate (Avanti^®^ Polar Lipids Inc., 810705). The TLC plate was developed in the solvent system containing diethyl ether/hexane/methanol/acetic acid (60:40:5:1, v/v/v/v) (Scot J. Stone personal communication). The newly synthesized fluorescent TAG was analyzed with a ChemiDoc MP Imager (Bio-Rad). Chemi-luminescent recombinant DGAT1s and fluorescent TAG was quantitated with the Image Lab software version 4.1 (Bio-Rad).

### Construction of diacylglycerol acyltransferase 1 expression vectors for *Camelina sativa*

The relevant DGAT1 coding sequence with a C-terminus V5-His tag were optimized for expression in Brassica species, synthesized by GENEART (Thermo Fisher Scientific) and sub cloned into pDONR™221. To simplify expression vector construction without altering the translated sequences we employed a similar strategy to that described above except *Xba*I instead of *Eco*RI was engineered at the 5′UTR. Chimeric DGAT1s were subsequently generated by exchanging the *Xba*I/*Xho*I digest fragment between the different parent constructs. The ΔN ZmL was constructed by substituting the *Xba*I/*Xho*I fragment of ZmL with one encoding for Met-Gly-Gly-Gly-Ser followed by the relevant 19 residues upstream of the conserved Leu-Ser-Ser sequence in the acyl-CoA binding domain.

A cassette consisting of *Not*I sites flanking the *Brassica napus* napin seed storage promoter region and 5′UTR (GenBank accession number EF627523.1):GATEWAY^^®^^ cloning sequences:octopine synthase terminator was synthesized by GenScript. The cassette was digested with *Not*I and cloned into pRSh1 (Scott et al., 2010) replacing the constitutive promoter cauliflower mosaic virus 35S (CaMV35Sp) driven GATEWAY^^®^^ adapted expression cassette. This created the binary vector pBR2 containing a seed specific expression cassette in a back-to back orientation with the CaMV35Sp driven *bar* gene for phosphinothricin resistant selection. The parent, ΔN ZmL DGAT1and chimera DGAT1s were subsequently placed into pBR2 from pDONR™221 by GATEWAY LR cloning (Thermo Fisher Scientific). The full length, chimeric and ΔN ZmL DGAT1 peptide sequences expressed in Camelina are listed in Supplementary Table 1.

### *Camelina sativa* growth conditions, transformation and selection

Seeds of *C. sativa* (cultivar Calena) were provided by the Field Service Center, Agriculture and Life Science Faculty, Lincoln University, Christchurch, New Zealand. Plants were grown in potting mix in a controlled climate room (16-h day length, 21–24°C, 65–70% relative humidity). To transform Camelina, flowers that were present approximately 5–6 weeks after planting were vacuum infiltrated (70–80 kPa abs for approximately 10 min) in a culture of *Agrobacterium tumefaciens* GV3101 cells harboring the appropriate expression construct re-suspended in a floral dip buffer (Clough and Bent, 1998). Plants were then kept for 24 h under low light conditions by partly covering with black plastic. Vacuum transformation was repeated 2–3 times on the same plant at 10–12 days intervals (depending on the duration of flowering). The subsequent T1 seeds were screened for herbicide resistance by germinating in continuous light, 22°C on half-strength Murashige and Skoog medium (pH 5.6) containing 1%(w/v) sucrose, 300 mg/L timentin, 25 mg/L DL-phosphinothricin, and 5.5 g/L gelrite. The majority of lines selected for analysis were single loci insertions identified by Southern probed with the *bar* gene.

### Crude protein extraction and purification of lipid droplets from *Camelina sativa*

To compare the accumulation of different recombinant DGAT1s in Camelina developing seeds were harvested at discreet developmental stages between 28 to 42 d after flowering, extracted, and analyzed by immunoblotting.

Lipid droplets (LDs) were extracted from 8 whole siliques in 1.5 mL extraction buffer (EB, 50 mM sodium phosphate buffer pH 7.2, 0.6 M sucrose, 150 mM NaCl, 1 mM PMSF and 1× protease inhibitor cocktail) (Roche 04 693 124 001) using an Omni-Bead Ruptor for two bursts of 30 s at a speed setting of 5.0. An 80 μL aliquot was taken from each crude extract and mixed with 20 μL of NuPAGE™ sample reducing agent (10×RdA, Thermo Fisher Scientific, NP009) and 100 μL of 2×LB (Winichayakul et al., 2013) and incubated at 37°C for 20 min. The remaining crude extract was centrifuged at 20,000 × *g* for 10 min at 4°C; the overlying immiscible LD fraction was transferred to a new tube, washed and centrifuged (1 mL EB, 20,000 × *g*, 4°C, 60 min × 2; then 0.5 mL EB, 20,000 × *g*, 4°C, 10 min × 3). Between each wash the aqueous layer and pellet were discarded. The purified LD fraction was re-suspended in 50 μL 2×LB, made up to total volume of 100 μL with EB, and incubated at 37°C for 20 min. Protein samples were separated and further subjected for immunoblot analysis.

### Lipid extraction and analysis

Total lipids from yeast cell DW were prepared by fatty acid methyl esterification (FAMEs) method as per Winichayakul et al. (2013). For quantification of total fatty acids in Camelina seeds, 20 seeds were weighed, homogenized and approximately 10 mg ground seeds were incubated in 1 mL of 1 M methanolic-HCl. The FAMEs samples were analyzed by GC-MS and quantified by way of internal standards.

To analyze TAG, lipids were extracted from approximately 15 mg of yeast cell DW according to Ruiz-López et al. (2003). The TAGs in 1 mL heptane containing 50 μg glyceryl trinonadecanoate TAG standard (Sigma T4632) were separated from the other lipid classes by Si-SPE column (Strata^®^ SI-1 Silica, 55 mm, 70Å). The column was conditioned with 2 mL methanol and equilibrated with 1.5 mL heptane before passing the TAG sample through. The flow-through fraction was collected in the glass tube. A further 1.2 mL heptane was passed through the column followed by 2 mL chloroform:heptane (1:9); the eluent was collected in the same glass tube. The flow-through TAG samples were dried under a stream of N2 and analyzed by FAMEs, and GC-MS (Winichayakul et al., 2013).

### Statistical analysis

Experiment data were analyzed by Student’s two-tailed *t*-test and one-way ANOVA using the RStudio version 3.6.0 with a model that included fixed effect of DGAT1 and controls (WT, VC, and null-sibling). A multiple comparison of treatments such as Bartlett’s test (homogeneity of variances) and Shapiro-Wilk normality test from ANOVA was used to highlight significant among treatment means while *P*-values were adjusted by the BH method (Benjamini and Hochberg, 1995) to control the false discovering rate. Means and SE are reported, and fixed effects declared significant from *P* < 0.05.

## Results and discussion

### The Poaceae have two distinct diacylglycerol acyltransferase 1’s

**FIGURE 1:**
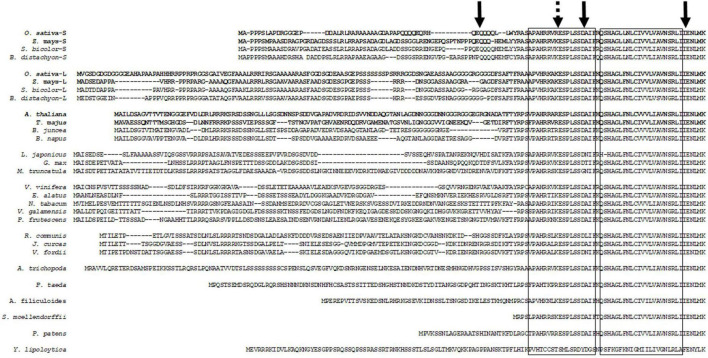
Alignment of representative plant DGAT1 N-terminal cytoplasmic regions and predicted first transmembrane domain. A number of these sequences are putative. Left hand box indicates acyl-CoA binding domain (Nykiforuk et al., 2002), right hand box indicates predicted first transmembrane domain (McFie et al., 2010). Left hand solid arrow indicates N-terminal truncation site used in this study and 3′ end of the Poaceae family DGAT1 GC compositional gradient. Dashed arrow indicates engineered *Xho*I restriction/ligation site used to generate chimeric DGAT1s. Center solid arrow indicates exon1-exon2 boundary, right hand solid arrow indicates exon2-exon3 boundary. Bold face species name and sequences indicate peptides used in this study. Accession numbers as follows: *A. trichopoda* (XM_006853832.3); ***A. thaliana***, NP_179535; *A. filiculoides* (Azfi_s0008.g011645); *B. distachyon* (long), XP_003568769; *B. distachyon* (short), XP_003560655; *B. juncea*, AAY40785; *B. napus*, AAD45536; *E. alatus*, AAV31083; *G. max*, NP_001237289; *J. curcas*, NP_001292926 and XP_012076954; *L. japonicas*, AAW51456; *M. truncatula*, XP_003595231; *N. tabacum*, NP_001313014 and XP_016503808; ***O. sativa*** (long), XP_015639406; ***O. sativa*** (short), XP_015641041; *P. frutescens*, AAG23696; *P. patens*, XP_001770929; *Pinus taeda* (1c1| PgdbPtadea_13664 congenie.org); *R. communis*, NP_001310663 and XP_002514132; *S. moellendorffii*, XP_002964165; *S. bicolor* (long), XP_002439419; *S. bicolor* (short), KXG20206; ***T. majus***, AAM03340; *V. fordii*, ABC94471; *V. galamensis*, ABV21945; *V. vinifera*, XP_002279345; *Y. lipolytica*, XP_50227; ***Z. mays*** (long), XP_008648150; ***Z. mays*** (short), NP_001288553 and XP_008649146.

The variable portion of the plant DGAT1 N-terminus is cytosolic and consists of a hydrophilic head of variable length which was recently determined to be an intrinsically disordered region (Caldo et al., 2017). Downstream of this is a relatively short region (12–13 residues) of increased similarity then a highly conserved acyl-CoA binding site (spanning exons 1 and 2) previously identified by Nykiforuk et al. (2002) (Figure 1). The variable N-terminal regions of the lycophyte and moss were relatively short compared to the DGAT1s from the vascular plants, it should be noted however, that we only had single examples of conifer, fern, lycophyte (primitive vascular) and moss (non-vascular). Phylogenetic analysis of plant DGAT1 peptide sequences encoded by exon 1 grouped them into clades corresponding with their taxonomic family where the Poaceae DGAT1s formed two discreet clades (referred to here as short and long) and each grass species had a representative in both clades (Supplementary Figure 1). The N-terminal hydrophilic encoding regions of the Poaceae DGAT1s were also found to contain a high GC compositional gradient along the direction of transcription (Supplementary Figure 2A); this is reportedly common at the 5′ end of Poaceae genes (Carels and Bernardi, 2000; Wong et al., 2002) and was speculated to be formed by local patterns of recombination (Glémin et al., 2014). Hidden Markov Model analysis with a biased early exit probability further revealed these contained varying numbers of direct and inverted repeats leading to a long and a short form of DGAT1 in each species (Supplementary Figure 2B). To our knowledge this is the first report of repeats within these GC compositional gradients. The two Poaceae DGAT1 clades presumably arose as a consequence of ancestral genome duplication (Salse et al., 2008) and subsequent genome diversification; their grouping into separate clades may indicate the diversification resulted in subfunctionalization (reviewed by Kellogg, 2003). Moreover, since the variable portion of the angiosperm DGAT1s is confined to the first exon, Siloto et al. (2010) speculated that this may have been used as an evolutionary mechanism to delimit the variability.

### Expression of recombinant diacylglycerol acyltransferase 1’s in *S. cerevisiae*

To investigate the role of the divergent cytosolic N-terminus we expressed full length and N-terminally truncated (ΔN) DGAT1s from *Arabidopsis thaliana* (At), *Tropaeolum majus* (Tm) as well as both short and long forms of *Oryza sativa* and *Zea mays* (OsS, OsL, ZmS, and ZmL, respectively) in yeast cells. The truncation was located 13 residues upstream of the conserved acyl-CoA binding region, this coincided with the carboxyl end of the divergent N-terminus and in the Poaceae family DGAT1s it also marked the 3′ end of the compositional gradient (Figure 1). Additionally, we generated reciprocal chimeras between the DGAT1s; these contained the hydrophilic variable N-terminal region and the first half of the conserved cytosolic acyl-CoA binding site from one DGAT1 and the remaining C-terminus from another. Given the high number of chimeric constructs we present only a subset of these, Tm:ZmS; ZmS:Tm; Tm:ZmL; ZmL:Tm; At:ZmL; ZmL:At, where the parent DGAT1 of the N-terminus is listed first followed by the parent DGAT1 of the C-terminus.

**FIGURE 2:**
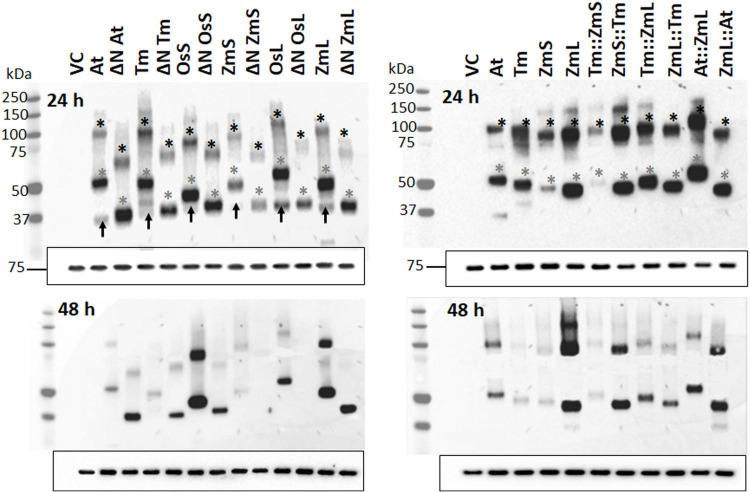
Immunoblot analysis of recombinant DGAT1s located in the microsomes from *Saccharomyces cerevisiae*. Immunoblot analysis of microsomal proteins extracted from 24 (top panels) and 48 h cultures (bottom panels); exposure time of the latter was approximately 10 times longer than those on the top. Gels were loaded based on equal quantities of Kar2 reference protein (Rose et al., 1989) shown by the 75 kDa band at the bottom of each immunoblot (similar results were obtained when the immunoblots were loaded on equal levels of total microsomal protein, not shown). Gray asterisks indicate recombinant DGAT1 monomers; expected monomeric sizes (kDa): At (62.7), ΔN At (52.9), Tm (62.6), ΔN Tm (53.3), OsS (58.9), ΔN OsS (54.1), ZmS (60.1), ΔN ZmS (53.7), OsL (63.4), ΔN OsL (53.3), ZmL (61.3), ΔN ZmL (53.3), Tm:ZmS (63.1), ZmS:Tm (59.6), Tm:ZmL (62.8), ZmL::Tm (61.0), At::ZmL (63.2), and ZmL::At (60.9). Black asterisks indicate dimerized DGAT1s. Black arrows in the top left-hand panel indicate C-terminal fragments from full length DGATs that have been autonomously N-terminally cleaved by *S. cerevisiae*. From the adjacent lanes the position of the autonomous cleavage appears to be very close to the ΔN position we selected. VC = Vector Control.

The use of the GAL1 promoter in *S. cerevisiae* normally results in a decline of recombinant protein at stationary phase (West et al., 1984; Shen et al., 2012); nevertheless, we were able to see that the N-terminus of DGAT1 influenced the long-term accumulation of both recombinant protein and lipids (Figure 2; Table 1; and Supplementary Table 2). Figure 2 showed that the recombinant DGAT1s migrated 15–20% faster in SDS-PAGE than their predicted molecular weights, the phenomenon was referred to as “gel shifting” by Rath et al. (2009) and is common for membrane proteins courtesy of their hydrophobicity (Shirai et al., 2008). When probed with the anti-V5 antibody many of the yeast cell protein extracts had multiple small discreet immunoreactive bands indicating the presence of recombinant C-terminal DGAT1 fragments which likely represent degradation intermediates of the protein. The pattern of these fragments was specific for each DGAT1 and appeared to be similar for the ΔN and respective full-length proteins suggesting the N-terminus was generally not involved (Supplementary Figure 3A). Possible exceptions to this were the chimeras Tm:ZmL and At:ZmL which had C-terminal fragments that were not present in cells expressing either the full length ZmL or its ΔN form; signifying that in some scenarios the N-terminus influenced the fragmentation (Supplementary Figure 3B). However, the majority of C-terminal fragments were not seen in the microsomal protein fraction suggesting in the total cell extract they likely represent incorrectly targeted/processed DGAT1 at various stages of degradation.

**TABLE 1:** Influence of expressing full length DGAT1s, ΔN DGAT1s and chimeric DGAT1s in *Saccharomyces cerevisiae* (quadruple mutant strain H1246, Sandager et al., 2002) on: cell growth (g DW/L); FA content (% DW); FA accumulation rate between 8–48 h (% FA/g DW/h); TAG accumulated at 48 h (% TAG/g DW).

Construct (*n* = *4)*	Cell growth			Lipid accumulation				
	g DW/L (8 h)	g DW/L (24 h)	g DW/L (48 h)	FA (% DW) (8 h)	FA (% DW) (24 h)	FA (% DW) (48 h)	FA (% DW)/h	TAG (% DW) (48 h)
Vc	0.71	5.3	4.6	2.5	2.4	3.2	0.02	0.0
At	0.45	3.8	4.6	5.6	5.2	5.4	0.00	1.3
Tm	0.55	4.8	5.7	5.1	5.7	7.1	0.05	2.5
OsS	0.64	5.5	6.0	4.4	6.2	8.5	0.10	3.8
ZmS	0.67	4.9	5.5	5.1	5.4	6.6	0.04	2.1
OsL	0.71	5.6	6.2	4.2	6.4	9.3	0.13	3.7
ZmL	0.72	6.3	6.9	4.0	6.6	8.9	0.12	4.4
ΔN At	0.32*	4.7**	5.8**	4.8	5.6	6.7*	0.05	1.9*
ΔN Tm	0.49	5.2	5.9	4.8	5.4	6.8	0.05	2.7
ΔN OsS	0.40**	4.8	5.6	4.9*	5.3**	6.3**	0.04	2.1***
ΔN ZmS	0.44*	4.6	5.0	5.2	4.8	5.2**	0.00	1.2***
ΔN OsL	0.72	6.0	6.9	5.0**	7.2	9.7	0.12	4.3*
ΔN ZmL	0.66	5.7	6.8	4.9**	7.8**	11.5**	0.17	5.4***
At::ZmL	0.38^#,∧∧∧^	2.8^##,∧∧∧^	4.4^∧∧∧^	3.5^###,∧^	3.6^###,∧∧∧^	3.7^##,∧∧∧^	0.00	0.3^##,∧∧∧^
ZmL::At	0.63^∧^	6.1^∧∧∧^	7.0^∧∧∧^	4.8^#^	6.6^∧∧^	9.3^∧∧∧^	0.11	4.9^#,∧∧∧^
Tm::ZmS	0.44^#,∧^	5.2	5.8	5.4	4.0^#^	5.6^##,∧∧^	0.00	1.6^###,∧^
ZmS::Tm	0.63	5.6^#,∧^	6.1	4.2∧	5.8	8.1^#^	0.10	3.1^#^
Tm::ZmL	0.48^∧∧^	5.1^∧∧^	6.4	5.3^∧^	6.3	8.5^##^	0.08	4.0^###,∧^
ZmL::Tm	0.67	5.9^∧^	6.8^∧∧^	4.1^∧^	5.9^#^	8.7^∧∧^	0.12	4.1^#,^ ∧∧∧

Significance difference relative to the full-length parent DGAT1 indicated by *, **, and *** represents P < 0.05, P < 0.01, and P < 0.001, respectively; significance difference relative to the N-terminal parent DGAT1 indicated by ^#^, ^##^, and ^###^ represents P < 0.05, P < 0.01, and P < 0.001, respectively; significant difference relative to the C-terminal parent DGAT1 indicated by ^∧^, ^∧∧^, and ^∧∧∧^ represents P < 0.05, P < 0.01, and P < 0.001, respectively. Standard errors are presented in Supplementary Table 2.

We observed that phylogenetically related DGAT1s behaved similarly, where after 48 h cells expressing ΔN At and ΔN Tm accumulated more recombinant protein and TAG than cells expressing their full-length versions; the opposite was true for ΔN OsS and ΔN ZmS. Interestingly, N-terminal truncation of both OsL and ZmL resulted in less recombinant protein but increased TAG content (Figure 2; Table 1; and Supplementary Table 2). It should be remembered that both OsL and ZmL are predicted to have highly positively charged N termini in extraction buffer; while over the same region and pH range the other DGAT1s are predicted to have almost no charge or a negative charge (Supplementary Table 3). It is not known if any of these differences have an influence on DGAT1 accumulation or extraction efficiency but may need to be considered to understand these specific results. Chimeras with the At or Tm N-terminus accumulated less recombinant protein and TAG than cells expressing the respective full-length parent (ZmL or ZmS) of the C-terminus; whereas the reciprocal chimeras accumulated more (Figure 2; Table 1; and Supplementary Table 2).

The comparable behavior of similar DGAT1 N-terminal regions was also noted previously where the four DGAT1s from *Brassica napus* were studied (Greer et al., 2015). Two originate from the Brassica A genome and two from the Brassica C genome; these grouped into two clades with the A and C genomes contributing one to each clade. Although all four *B. napus* DGAT1s are relatively similar, when reciprocal chimeras were generated using the first exon from one clade and the remainder of the sequence from the other, the quantity of TAG and recombinant protein that accumulated was similar to the DGAT1 that contributed the N-terminus.

Curiously, the N-terminus of the recombinant DGAT1s also appears to influence the quantity of total microsomal proteins in the yeast cells. Cells expressing ZmL accumulated 40–50% less total microsomal protein than ΔN ZmL, At:ZmL or Tm:ZmL (Supplementary Figure 4). However, At and Tm both accumulated approximately the same quantity as ZmL:At and ZmL:Tm, respectively.

### A portion of the recombinant diacylglycerol acyltransferase 1 is found in the lipid droplet

Although DGAT1 has been shown to be an integral membrane protein of the ER (Shockey et al., 2006; McFie et al., 2010) DGAT1 activity has been reported to be associated with lipid droplets (LDs) and was thought to be due to the DGAT1 remaining in the LDs after it has pinched off from the ER (Lung and Weselake, 2006). When we performed immunoblot analysis on the fat pad and LDs from yeast and Camelina, respectively, we also found recombinant DGAT1s were present (Supplementary Figure 5). However, DGAT1 from in the Camelina LDs electrophoresed only as large oligomers. It is not known if these were homo- or hetero-oligomers.

### The plant diacylglycerol acyltransferase 1 C-terminus may have a similar topology to the mammalian orthologs

Given the different reported topologies of the plant and animal DGAT1s (Shockey et al., 2006; McFie et al., 2010; Chen et al., 2017) we combine N- and C-terminal as well as internal tags with high pH membrane reformation, protease protection and differential permeabilization to determine if the orientation of the recombinant plant DGAT1 in the yeast ER was comparable to either of the proposed forms. We performed protease protection assays on microsomal preparations from cells expressing ΔN, full length and chimeric DGAT1s (all with the C-terminal V5 epitope). In each case the V5 tag was still detected but as smaller fragments which indicates the C-terminus is located in the lumen. Moreover, the smaller peptide fragment pattern was specific for each C-terminus and appeared to be predominantly a mixture of monomers and one or two differentially electrophoresing dimers (Supplementary Figure 6).

**FIGURE 3:**
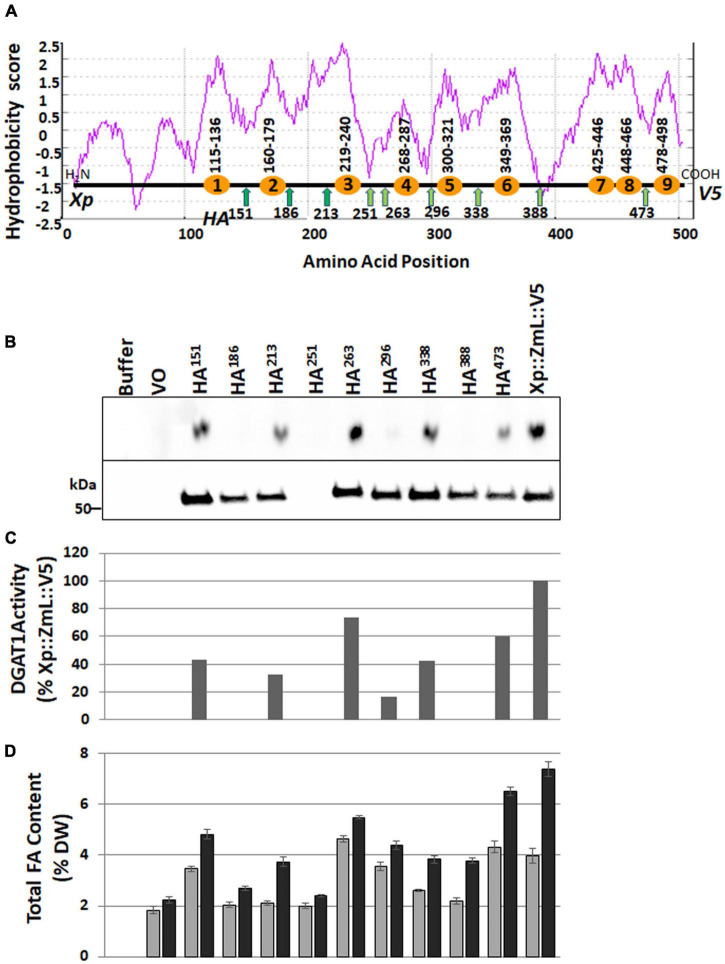
Schematic representations of ZmL DGAT1 and influence of internal HA epitope tags on accumulation of recombinant ZmL DGAT1 protein, specific activity and total lipid content. **(A)** Hydrophobicity score aligned with the position of the TMpred (https://sbcb.bioch.ox.ac.uk/, Hofmann and Stoffel, 1993) predicted transmembrane domains are shown as orange circles; the number range above each circle indicate the predicted first and last residues of the domain. The positions of the engineered internal HA tags are shown as green arrows, the number below each arrow is the position of the N-terminal residue of the tag (only one HA tag was used per construct). Xp represents the X-press tag and V5 represents the C-terminal tag. **(B)** The upper panel shows the amounts of triacylglycerol produced by the corresponding recombinant DGAT1. Equivalent quantities of microsomal protein were used in an *in vitro* DGAT1 activity assay. The lower panel shows an immunoblot that had been loaded with equivalent quantities of microsomal protein from 24 h yeast cultures and probed using the anti V5 antibody. No recombinant protein was detected in the culture expressing Xp::ZmL-HA^251^::V5 (upper panel). **(C)** The relative specific activity of the internally HA tagged ZmL constructs. The results were adjusted for the level of recombinant DGAT1 (after the V5 signals were quantified with Image Lab Software, V4.1, Bio-Rad) in the microsomal preparations and the quantities of TAG were then normalized to the quantity of TAG produced by the Xp::ZmL::V5. **(D)** The total FA content of the yeast cultures as a% of cell DW after 24 (gray bars) and 48 h (black bars) of growth.

Downstream of the variable N-terminus the hydrophobicity plots of each plant DGAT1s was similar; and the number of transmembrane helices was predicted by TMpred (Hofmann and Stoffel, 1993) to be between 8–10 (Figure 3A). Given the overall similarities we chose to further investigate the number and position of the transmembrane domains by focusing on one DGAT1, ZmL. Subsequently we generated 10 variants, nine of these contained three tags each: an N-terminal X-press epitope, a uniquely positioned internal HA epitope, and a C-terminal V5 epitope, the tenth variant (XP:ZmL:V5) had the N- and C-terminal tags but no internal HA tag. The nine internally tagged constructs were labeled according to the position of the N-terminal residue of the HA epitope (XP:ZmL-HA^151^:V5, XP:ZmL-HA^186^:V5, XP:ZmL-HA^213^:V5, XP:ZmL-HA^251^:V5, XP:ZmL-HA^263^:V5, XP:ZmL-HA^296^:V5, XP:ZmL-HA^338^:V5, XP:ZmL-HA^388^:V5, and XP:ZmL-HA^473^:V5) which were all located between predicted transmembrane domains (Figure 3A). The constructs were individually expressed in *S. cerevisiae* (strain H1246) and subsequently analyzed for DGAT activity, accumulation of the recombinant DGAT1, and FA content (Figures 3B–D). The internally tagged DGAT1s were shown to be functional by the accumulation of lipids in *S. cerevisiae*. However, it should be noted that the specific activity, accumulation of the recombinant DGAT1, and FA was reduced compared to XP:ZmL:V5. Furthermore, the recombinant XP:ZmL-HA^251^ was not detected.

**FIGURE 4:**
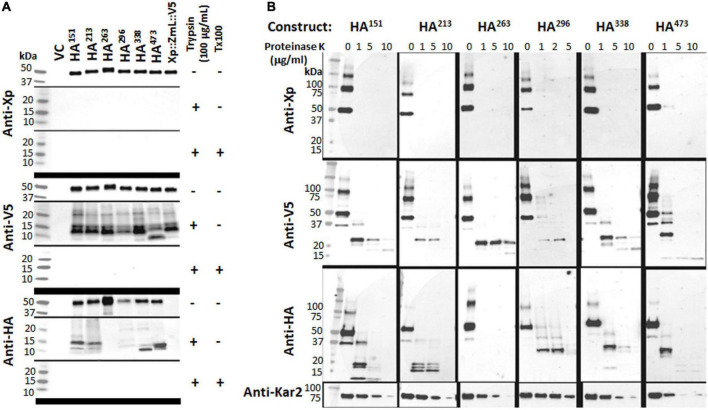
**(A)** The addition of trypsin alone resulted in the total disappearance of the Xpress (Xp) tag and the appearance of conserved fragments containing the V5 tag; when both trypsin and triton X-100 were added the V5 signal was also lost. **(B)** The addition of proteinase K produced similar results. Combined, the data indicate the N-terminus of each tagged DGAT1 is located in the cytoplasm and the C-terminus is located in the lumen. In contrast to the N- and C-terminal tags, the susceptibility of the internal HA tags to protease was dependent on the construct. The addition of trypsin alone or proteinase K resulted in the loss of the HA signal from Xp::ZmL-HA^263^::V5 (HA^263^), whereas HA^151^ and HA^213^ all had similar conserved fragments while HA^296^, HA^338^, and HA^473^ had a different set of conserved fragments; no HA was detected from any construct after the addition of both trypsin and triton X-100. Thus, residues 151–213 appear to belong to the same luminal loop, residue 263 is located in the cytoplasm, and from residue 296 to the C-terminus are located between the integral membrane to the lumen. The protease protection assays for HA^251^ have not been considered as no recombinant protein was detected. The yeast KAR2 protein is most homologous to mammalian BiP/GRP78 and was detected in this study as a control for the ER-localized protein. VC, vector control.

Microsomal preparations from the cultures of XP:ZmL-HA^151^:V5, XP:ZmL-HA^213^:V5, XP:ZmL-HA^263^:V5, XP:ZmL-HA^296^:V5, XP:ZmL-HA^338^:V5 and XP:ZmL-HA^473^:V5 were sequentially subjected to protease protection assays using trypsin. The protease protection assays for XP:ZmL-HA^186^:V5, and XP:ZmL-HA^388^:V5 have not been considered as no DGAT1 activity was detected. In each case the addition of trypsin alone resulted in the complete disappearance of the XP tag, whereas the V5 signals were replaced with conserved faster migrating fragments (Figure 4A). In comparison the susceptibility of the HA tag to trypsin was dependent on the construct, where the signal was lost from XP:ZmL-HA^263^:V5. Constructs XP:ZmL-HA^151^:V5, and XP:ZmL-HA^213^:V5 all produced comparable fragments, while XP:ZmL-HA^296^:V5, XP:ZmL-HA^338^:V5 and XP:ZmL-HA^473^:V5 had a different group of fragments. All Xpress, V5 and HA signals disappeared with the addition of both trypsin and TX-100 (Figure 4A).

Similar to trypsin, the use of PNK in the protease protection assay resulted in the disappearance of the XP tag, while the V5 tag remained (Figure 4B). Similar groups of differentiate fragments of the HA signals were observed with the PNK digestion and were specific for each HA tag (Figure 4B). A range of PNK concentrations were used (1, 5, and 10 μg/ml) and while the XP tag was no longer detected at the lowest concentration, the V5 and Kar2 (ER luminal control) signal intensities decreased with increasing PNK concentration. The latter indicates that the microsomes were not completely sealed. The V5 signals were predominantly detected as a single smaller fragment that was fractionally larger for XP:ZmL-HA^473^.

**FIGURE 5:**
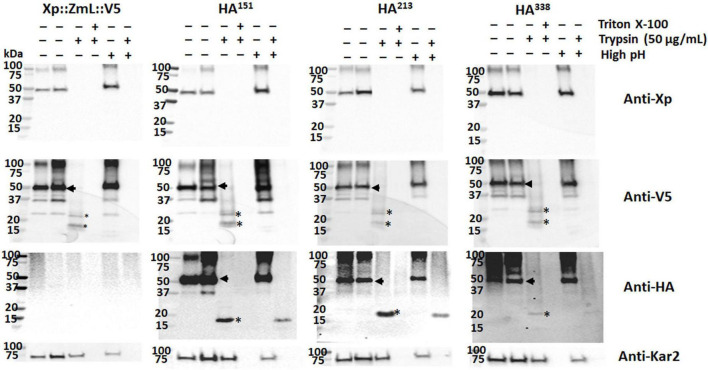
Sequential high pH membrane reformation/protease protection assay of microsomes containing DGAT1. High pH conditions reformed the sealed microsomal compartment, allowing protease cleavage of luminal domains from integral domains embedded in the membrane bilayers (Wu et al., 2003). The addition of trypsin alone resulted in the total disappearance of the Xpress (Xp) tag and the appearance of conserved fragments containing the V5 tag; when both trypsin and triton X-100 were added the V5 signal was also lost. Combining high pH treatment with trypsin digestion resulted in the disappearance of the V5 tag of all Xp::ZmL::V5, Xp::ZmL-HA^151^::V5 (HA^151^), HA^213^, and HA^338^. The data indicates the C-terminus is located in the lumen. In contrast to the C-terminal tags, although the addition of trypsin after high-pH conditions resulted in the reduction of the HA signal in all HA^151^, HA^213^, and HA^338^, these conserved fragments were visible. Thus residues around 151, 213, and 338 appear to belong to the integral membrane. Arrows indicate full-length DGAT1s, * indicated fragmentation of DGAT1s after protease digestion.

Exposure of sealed membrane structures to high pH reforms the integrity of the membrane without denaturing the lipid bilayer (Wu et al., 2003). Microsomal preparations from the cultures of XP:ZmL:V5, XP:ZmL-HA^151^:V5, XP:ZmL-HA^213^:V5 and XP:ZmL-HA^338^:V5 were subjected to combinations of detergent (TX-100), high pH and protease digestion. This allowed cleavage of both cytoplasmic and luminal domains from the integral membrane portions of the protein. In all cases, the addition of trypsin to the high pH reformed membranes resulted to the complete disappearance of the smaller V5 signal whereas the HA signals of XP:ZmL-HA^151^:V5, XP:ZmL-HA^213^:V5 and XP:ZmL-HA^338^:V5 were remained, indicating these were located within the membrane (Figure 5).

**FIGURE 6:**
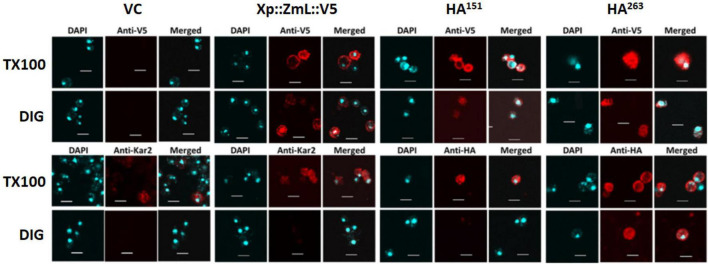
Differential permeabilization analysis of *S. cerevisiae* cells expressing VC, Xp::ZmL::V5, Xp::ZmL-HA^151^::V5 and Xp::ZmL-HA^263^::V5. Nuclei of individual cells were visualized by DAPI stain. As expected, the ER lumen marker protein Kar2 was visualized after treatment with triton X-100 (TX100) but not digitonin (DIG). After treatment with DIG and probing with the V5 antibody the majority of cells had a faint signal; however, the signal was much stronger when the cells were treated with TX100. The HA signal on HA^151^ was not detected when the cells were pre-treated with DIG but was detected after pre-treatment with TX100 whereas the HA signal on HA^263^ was detected after the addition of either detergent. Combined, the results concur with the protease protection assay and indicate the C-terminus and the internal residue 151 on ZmL DGAT1 are located in the membrane compartment while the internal residue 263 is located in the cytoplasm. Scale bar = 5 μM.

**FIGURE 7:**
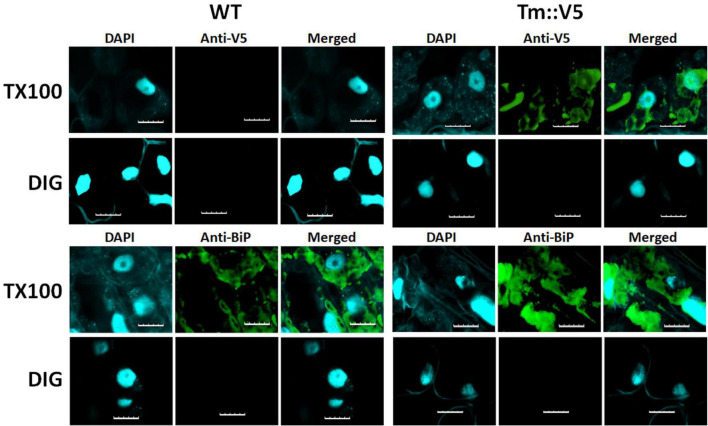
Differential permeabilization analysis of *L. perenne* cells expressing Tm DGAT1. Nuclei of individual cells of wild-type (WT) and cells expressing C-terminally V5 tagged recombinant *T. majus* DGAT1 (Tm::V5) were visualized by DAPI stain. Cells were permeabilized with either digitonin (DIG) or triton X-100 (TX100) then probed with antibodies raised against either V5 or Bip, followed by the appropriate secondary antibody. No V5 or Bip signal was detected when cells were permeabilized with DIG. However, both V5 and Bip were detected in cells expressing Tm after they were permeabilized with TX100 indicating the C-terminus of Tm DGAT1 is located in the membrane compartment. Scale bar = 10 μm.

In addition to protease protection assays we used differential permeabilization to study recombinant DGAT1 topology in both *S. cerevisiae* and *Lolium perenne*. The nuclei of both *S. cerevisiae* and *L. perenne* were visualized by DAPI stain; while Kar2 (Rose et al., 1989) and the binding protein BiP (Gething, 1999) served as ER luminal marker proteins for yeast and *L. perenne*, respectively. Following DIG treatment, we detected a faint V5 signal in the bulk of yeast cells expressing the DGAT1s with the N-terminal X-press, internal HA and C-terminal V5 epitopes (Figure 6). However, the signal was much stronger when the cells were treated with TX100 indicating that the majority of C-termini were located in the lumen (Figure 6). Since we previously found the whole cell protein extracts (but not microsomal preparations) from *S. cerevisiae* contained multiple small fragments of the C-terminus (which were presumed to belong to incorrectly targeted/processed DGAT1 at various stages of degradation) we suggest these may explain the faint V5 signal seen in cells treated with DIG. The Kar2 and HA signals (from XP:ZmL-HA^151^:V5) were detected after permeabilization with TX100 but not DIG (indicating they are located in the lumen). In comparison, the HA tag from XP:ZmL-HA^263^:V5 was detected after permeabilization with DIG signifying it is located in the cytoplasm. In *L. perenne*, neither Bip nor the C-terminal V5 tag of Tm were detected after permeabilization with DIG. Instead, both were detected only after permeabilization with TX-100, indicating that *in planta* the C-terminus of Tm DGAT1 is also located in the lumen (Figure 7). Thus, the predicted topology of the plant C-terminal DGAT1s was the same whether determined by protease protection assay or differential permeabilization. Recently, Wurie et al. (2011) suggested that DGAT1 may have a dual topology which could explain why there appears to be a difference between the topologies of the DGAT1s (even though their hydrophobicity plots are very similar). In our case it could be argued that the combination of a faint V5 signal seen by confocal microscopy when the yeast cells were permeabilized with DIG and the small loss of the V5 signal when the microsomal membrane preparations were incubated with trypsin may be due to a dual topology. However, it would need to be determined if the apparent dual topology is a reflection of what exists in nature or is the result of heterologous expression of the protein.

**FIGURE 8:**
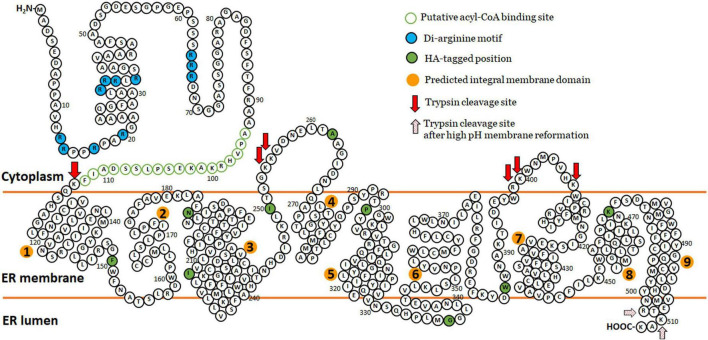
The predicted topology model of maize ZmL DGAT1. Combining those predicted models of TmPred and AlphaFold and our results from protease protection/internal HA-tag strategy similar to McFie et al. (2010) and high pH reformed membrane compartments (Wu et al., 2003) we speculate nine transmembrane domains (orange circles) between the two termini which create two cytoplasmic loops (at least residue 262–270 and at least residue 398–406), and a C-terminal tail in the lumen (consisting of at least residue 500 to the C-terminus). Our results from the protease digestion of HA^296^ suggested these residues around 296 is not exposed into cytosol. The original amino acid sequence of ZmL DGAT1 (XP_008648150) is represented by black circles. HA tag position is represented by black circles filled with green. The putative acyl-CoA binding site is presented by green circles and the putative di-arginine motifs are presented by black circles filled with turquoise. Red and red dotting arrows indicated estimated positions cleavable by trypsin digestion.

Collectively, the protease protection results indicate that in *S. cerevisiae* the N-terminus of ZmL is located in the cytoplasm and the C-terminus is luminal. The topology of the DGAT1s has not been absolutely elucidated, for example, modeling predicted that tung tree DGAT1 contains 10 transmembrane domains (Shockey et al., 2006) and that the mouse DGAT1 would contain eight (McFie et al., 2010). However, protease protection combined with constructs containing internal tags demonstrated that the mouse DGAT1 has only three transmembrane domains and that the C-terminus is located in the lumen (McFie et al., 2010). In comparison differential permeabilization was used to determine that the C-terminus of tung tree DGAT1 was cytoplasmically located (Shockey et al., 2006). For ZmL, Tmpred modeling predicted nine transmembrane domains (Figure 3A; Hofmann and Stoffel, 1993). The overall three-dimensional predicted structure of the ZmL DGAT1 modified from AlphaFold^3^ indicates at least nine α-helix structure of hydrophobic domains with exposed N- and C-termini in opposite directions (Supplementary Figure 7). Combining these predicted models and our results from protease protection/internal HA-tag, high pH membrane reformation and differential permeabilization we speculate nine integral membrane domains between the two termini which create two cytoplasmic loops (at least residue 262–270 and at least residue 398–406), and a C-terminal tail in the lumen (consisting of at least residue 500 to the C-terminus) (Figure 8). The results from the protease digestion of XP:ZmL-HA^296^:V5 suggested the residues flanking residue 296 are not exposed into cytosol. However, it should be noted that assignment of these assays is based on estimations of the cleavage fragments. Therefore, exact position of these loops, including integral and luminal loops was not determined due to the limited number of overall constructs. The confidence in the assignment is dependent on results from the HA tagged to their respective DGAT1 residues with the possibility remains that they are artificial. Therefore, a more comprehensive proteomic analysis such as multidimensional protein identification technology is further required to confirm the prediction sites (Wu et al., 2003; Wang et al., 2012; Lee and Kim, 2014).

### Expression of modified diacylglycerol acyltransferase 1 N-terminus increases lipid content in *C. sativa* seeds

Although the *S. cerevisiae* expression system is a comparatively rapid and convenient way to investigate the structure/function of recombinant eukaryotic proteins it may not always reflect the native environment of the protein under study. To more closely approximate this we over-expressed three full length DGAT1s (Tm; ZmS; and ZmL), one N-terminally truncated DGAT1 (ΔN ZmL), and four chimeric DGAT1s (Tm:ZmS; ZmS:Tm; ZmL:Tm; and Tm:ZmL) in the developing seeds of *Camelina sativa* under the control the *Brassica napus* 1.7 S storage protein (napin) promoter (Josefsson et al., 1987). All constructs were optimized for expression in Camelina; in addition, the putative serine/threonine protein kinase site in the *Tropaeolum majus* DGAT1 (Xu et al., 2008) was disrupted by substitution of the serine to alanine, generating Tm^S197A^; ZmS:Tm^S170A^; and ZmL:Tm^S189A^. Homozygous T3 seeds and their respective null segregants were analyzed to determine the FA profile, seed size and lipid content. Of the lines expressing a standard full length DGAT1 only Tm#2 and ZmL#9 (from 3–4 lines identified from each parent construct) had significantly more lipids per seed (% seed lipid and/or mg lipid/seed) than the wild type and the appropriate null sibling (Table 2 and Supplementary Tables 4, 5). In comparison, the majority of the lines expressing ΔN ZmL (2 lines) or any of the chimeric DGAT1s (2–4 lines identified from each construct) had highly significant (*P* < 0.001) increases in lipid levels compared to both wild type and their respective null siblings (Table 2 and Supplementary Tables 4, 5). Furthermore, at least one line from each of the chimeras had a significant increase in seed size.

**TABLE 2:** Comparison of seed weight (mg), seed lipid content (% DW), and mg lipid per seed in wild-type (WT) and vector control (VC) as well as individual lines of homozygous (HOM) and respective null sibling *Camelina sativa* seeds from plants that had been transformed with either a full length DGAT1, a ΔN ZmL or a chimeric DGAT1.

Plant (*n* = *8*)	HOM seed size (mg/seed)	Null seed size (mg/seed)	HOM seed lipid (%)	Null seed lipid (%)	HOM Lipid/seed (mg/seed)	Null Lipid/seed (mg/seed)
WT	1.05		27.3		0.288	
VC	1.05		26.5		0.278	
Tm#2	1.05	1.08	29.5*,^#^	26.2	0.315	0.293
Tm#5	0.99*	1.00	28.8	26.5	0.288	0.265
ZmS#1	1.01	1.08	26.2	26.2	0.265	0.284
ZmS#18	1.01*	1.02	28.0	28.1	0.281	0.287
ZmL#6	0.84***	0.93**	26.4	27.6	0.231**	0.246**
ZmL#9	1.02	1.05	30.1**, ^###^	26.5	0.303^##^	0.277
ΔN ZmL#1	1.13**	1.08	31.4***^###^	26.5	0.345***,^###^	0.286
ΔN ZmL#2	1.03	1.04	31.6***, ^###^	28.1	0.326**, ^##^	0.295
Tm::ZmS#8	0.94**, ^#^	0.86	26.8	27.3	0.248	0.249
Tm::ZmS#9	1.04	0.99	32.5***, ^###^	25.6	0.337**, ^###^	0.260
Tm::ZmL#5	1.03	1.03	42.0***, ^###^	25.8	0.432**^*,###^	0.267
Tm::ZmL#13	1.16**, ^###^	1.04	37.2***, ^###^	25.4	0.432***, ^###^	0.264
ZmS::Tm#3	1.36***, ^###^	1.01	39.3***, ^###^	27.4	0.536***, ^###^	0.276
ZmS::Tm#4	1.51***, ^###^	1.01	33.9***, ^###^	26.2	0.513***, ^###^	0.264
ZmL::Tm#22	1.21***, ^###^	1.09	32.9***, ^###^	26.9	0.399***, ^###^	0.297
ZmL::Tm#23	1.04	1.03	27.3	26.5	0.275	0.276

Significant difference relative to the WT indicated by *, **, and *** represents P < 0.05, P < 0.01, and P < 0.001, respectively; significant difference relative to the null siblings indicated by ^#^, ^#^, and ^##^ represents P < 0.05, P < 0.01, and P < 0.001, respectively. Data from more independent lines are presented in Supplementary Table 4 and standard errors are presented in Supplementary Table 5.

**FIGURE 9:**
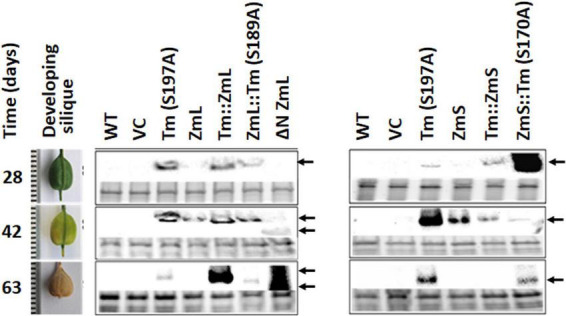
Accumulation of recombinant DGAT1s under the control of the *Brassica napus* 1.7 S storage protein napin promoter (Josefsson et al., 1987) in *Camelina sativa* developing siliques. The harvest intervals and associated developmental stages are shown in the left-hand panel. Recombinant DGAT1s were detected by immunoblots probed with anti V5 antibodies (center and right-hand panels). Gels were loaded on equal levels of protein shown by the Bio-Rad Stain-Free images for each lane in the lower portion of each boxed sampling date. Arrows indicate recombinant DGAT1 signal; double arrows at the last two sampling dates indicate the different electrophoretic mobility of ΔN ZmL compared to the full length and chimeric DGAT1s. It should be noted that in order to detect the recombinant DGAT1s in the tissue sampled 63 days after flowering these lanes were loaded with twice the amount of total protein extract than the earlier sampling dates.

Under our growth conditions there was approximately 5 days between inflorescence emergence and pollination of *C. sativa*, a further 37 days for the seeds to reach maximum size and then 21 days for the siliques to lose all green coloration as they desiccate (63 days in total). Accumulation of the majority of recombinant DGAT1s (Tm^S197A^, ZmS, ZmL, ΔN ZmL, ZmL:Tm^S189A^, and Tm:ZmS) in *C. sativa* seeds correlated with the expected transcript profile for constructs under the control of the *B. napus* napin promoter, where the *B. napus* storage protein transcript is highest between 32–45 days after pollination (DAP) and was either not detected or was at very low levels during early seed development in Canola (Hu et al., 2009). This time frame corresponds with the developing seed approaching maximum fresh weight through to the seed maturation stage (Borisjuk et al., 2013) and correlates with the last three stages we analyzed in Camelina, 28–63 days after flowering; or 23–58 DAP (Figure 9). However, compared to the parent DGAT1s, truncated ZmL and four chimeras were detected over a range of different developmental stages which may explain the increased lipid yields per seed.

In a comprehensive review of DGATs Liu et al. (2012) noted that the level of most oilseed DGAT1 transcripts are closely related with TAG accumulation. There were several exceptions, including: *B. napus* (Nykiforuk et al., 2002) and *Ricinus communis* (He et al., 2004) where it was concluded that the respective *DGAT1* genes were likely to be regulated at the posttranscriptional level. Similarly, the mammalian DGAT1s are also proposed to be controlled by posttranscriptional mechanisms where blockage of the proteolytic pathways suggested this is likely to be at the level of translation and not protein stability (Yu et al., 2002; Casaschi et al., 2005). Since our constructs utilized the same promoter and each had been optimized for both mRNA stability and translational efficiency it would suggest that variances in DGAT1 accumulation are likely due to post translational factors. To our knowledge there are no reports of the ubiquitin pathway being involved in DGAT1 degradation. Indeed, the mutation of a potential ubiquitination site and a potential phosphorylation site on *B. napus* DGAT1s had no effect on enzyme activity which led Greer et al. (2015) to conclude enzyme accumulation was not affected. Given the high degree of sequence variation of the N-termini, we cannot however, exclude the N-end rule pathway from being involved *in planta* (Graciet and Wellmer, 2010; Sriram et al., 2011; Tasaki et al., 2012). The changes we observed in accumulation of chimeras compared to both full length DGAT parents in *S. cerevisiae* and *C. sativa* suggests that if the N-end rule pathway is involved then it is not the only determining factor.

### Expression of modified diacylglycerol acyltransferase 1 N-terminus alters FA profile of *C. sativa* seeds

Expression of the recombinant DGAT1s also influenced the FA profile of the seed compared to the WT; there were numerous significant differences between the profile of each chimeric DGAT and both N-terminal and C-terminal parents (Table 3 and Supplementary Table 6). Of the 13 species of FAs quantified in the seeds of Camelina the proportions of 11 of them were significantly altered compared to WT depending on which DGAT1 was over expressed. This may reflect different substrate specificities of the recombinant DGAT1s. However, it could also point toward variances in both substrate and co-factor availability during the time the recombinant DGAT1s accumulate.

**TABLE 3:** FA profiles of controls and homozygous *Camelina sativa* seeds from plants that had been transformed with either a DGAT1, a ΔN ZmL or a chimeric DGAT1.

Plant (*n* = *8*)	FA species (as a% of total FA)												
	C16:0	C18:0	C18:1	C18:2	C18:3	C20:1	C20:2	C20:3	C22:0	C22:1	C22:3	C24:0	C24:1
Control	7.33	2.75	10.77	20.35	34.11	13.98	2.10	1.29	0.47	4.88	0.87	0.25	0.85
Tm	6.58**	3.08	9.21	20.10	32.27	18.54***	2.13	1.25	0.76**	3.95	0.74	0.59***	0.80
ZmS	6.58***	2.41	9.78	17.29**	36.28	15.33**	2.75***	1.98***	0.69**	4.56	0.95	0.63***	0.77
ZmL	5.93***	2.59	9.80	18.62	34.40	16.12***	1.97	1.51*	0.55	5.76	1.21**	0.35*	1.19*
ΔN ZmL	7.17	2.18*	9.31	16.8**	39.73**	14.42	2.27	1.88***	0.40	4.29	0.81	0.21	0.53*
Tm::ZmS	6.46***	2.92	10.98	16.82**	33.87	17.13***	2.42**	1.79***	0.71**	4.63	0.83	0.47***	0.97
Tm::ZmL	6.18***	2.66	10.12	19.16	31.05	20.03***	2.19	1.29	0.78***	4.07	0.77	0.9***	0.80
ZmS::Tm	6.15***	2.19***	11.02	16.78***	38.63***	14.67*	2.10	1.66***	0.36*	4.49	0.82	0.25	0.88
ZmL::Tm	6.53***	2.32	11.25	18.63	35.21	15.33**	2.03	1.49*	0.28*	4.75	1.26**	0.25	0.67

Significant difference relative to the control indicated by *, **, and *** represents P < 0.05, P < 0.01, and P < 0.001, respectively. Standard errors are presented in Supplementary Table 6.

Of the 13 FA quantified, C18:1 and C22:1 were not significantly altered compared with WT. Not only is C18:1 one of the main FAs of the seed (approximately 10% of total FA), it is also the predominant FA exported by the plastid (Shine et al., 1976; Browse et al., 1986; Somerville and Browse, 1991). Following export, C18:1 is condensed with CoA before it can be modified (desaturated; elongated) *via* a metabolic network involving the acyl editing cycle. The cycle uses phosphatidylcholine and lysophosphatidylcholine as support molecules to enable both the exchange of FA species as well as desaturation and other modifications of the FA in the *sn-2* position (Bates et al., 2009). In a schematic depiction of TAG biosynthesis in plants Chapman and Ohlrogge (2012) presented two pools of acyl-CoA with one associated with the plastid (containing C16:0, C18:0, and C18:1) and the second associated with the acyl editing cycle. They noted that labeling indicated the pools co-mingled, although separate pools of intermediates were also thought to exist. In our case, it could be useful to fully analyze all the species of TAGs in order to help determine how the relatively constant proportion of C18:1 is maintained.

### The N-terminus of diacylglycerol acyltransferase 1 influences oligomerization

**FIGURE 10:**
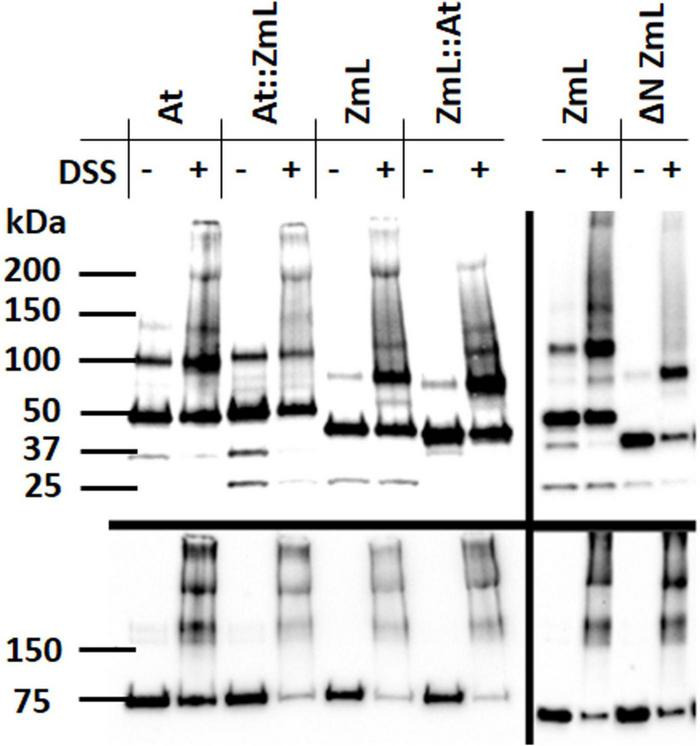
Influence of the N-terminus on DGAT1 oligomerization. Immunoblot analysis of microsomal proteins from 24 h cultures of *S. cerevisiae* expressing At, ZmL and the reciprocal chimeras At:ZmL and ZmL:At (left hand panels); ZmL and ΔN ZmL (right hand panels). Prior to protein extraction each microsomal preparation was divided into two aliquots; half were treated with cross linking agent disuccinimidyl suberate (DSS). The addition of DSS to the full length and chimeric DGAT1s resulted in the generation of higher order oligomers; however, these were absent in the ΔN ZmL sample treated with DSS. Upper panels were probed with anti-V5 antibody while lower panels were probed with anti-Kar2 antibody.

In the absence of the cross-linking agent DSS all forms of the DGAT1s (ΔN, full length and chimeras) electrophoresed in SDS-PAGE predominantly as both monomers and dimers; in some instances, larger oligomers (potentially trimers) were also detected (Figure 10). To explore if there was a relationship between oligomerization and the N-terminus we pre-treated yeast microsomal samples with DSS. This resulted in a decrease in the proportion of monomeric full length and chimeric DGAT1s and increases in the proportions of their dimers and higher oligomers. A reduction in the level of monomer was also seen following DSS treatment of ΔN ZmL; however, in this case the largest oligomer detected was a dimer (Figure 10). These data support the role of the N-terminus in oligomerization.

### Possible roles for the di-arginine motifs in the N-terminus

The greatest increases in accumulation of recombinant DGAT1 and lipids in Camelina seeds came from the chimeras ZmS:Tm^S170A^ and Tm:ZmL (Figure 9; Table 2; and Supplementary Tables 4, 5); given the differences in the length and sequence of their N-termini we are curious to know how this occurs and why only for these chimeras? Siloto et al. (2010) pointed out that DGAT1s from a broad range of organisms contain a cluster of arginines in the first 30 residues. Closer examination reveals the majority of angiosperm DGAT1s contain a diverse range of di-arginine motifs (RR, RXR, and RXXR) in the variable region of the N-terminus (Figure 1). In comparison, the mammalian DGAT1s (including: *Bos Taurus*, NP_777118; *Mus musculus*, NP_034176; *Homo sapiens*, NP_036211; *Ovis aries*, NP_0011036; *Rattus norvegicus*, NP_445889; *Sus scrofa*, NP_999216; and *Mesocricetus auratus*, XP_005086048) contain the same multi motif (RRRR) close to the N-terminus and a second motif (RXXR) at the start of the acyl-CoA binding region.

There are several proposed roles for N-terminal di-arginine motifs including targeting membrane proteins to the ER (Boulaflous et al., 2009) and determining topology (Parks and Lamb, 1993). However, in this study 24 h after induction, each full length and respective ΔN DGAT1 were found in comparable quantities in the microsomal preparations; suggesting that in the case of the plant DGAT1s we investigated the primary function of the di-arginine motifs is not ER targeting. Like the mammalian DGAT1s, a conserved di-arginine motif is also found in the N-terminus of the cytoplasmic acyl-CoA binding domain of At, Tm, OsS, and ZmS (and would therefore be present in the ΔN forms) but it is not present in OsL or ZmL. Interestingly, ΔN OsL and ΔN ZmL were also the two truncated DGAT1s that had less recombinant protein but more TAG after 48 h compared to their full-length counterparts.

We reported above that several of the recombinant DGAT1s were detected in the fat pad and LD fractions of *S. cerevisiae* and *C. sativa*, respectively (Supplementary Figure 4). The yeast fat pad DGAT1 may however be contamination since this fraction also showed the presence of the ER marker protein Kar2. In contrast, the Camelina LDs had no detectable ER marker protein BiP; moreover, the electrophoretic migration as large oligomers of the DGAT1 from the LDs supports the absence of ER lipid bi-layer contamination. Di-arginine motifs located close to the terminus of a cytoplasmic domain can be involved in retrieval of ER membrane proteins from the Golgi apparatus and the ER-Golgi intermediate (Teasdale and Jackson, 1996); multiple motifs were found to be more effective at retrieval than singles (Boulaflous et al., 2009). Since DGAT has been found on the LDs it would be interesting to determine if the di-arginine motifs can act as retrieval signals from the LDs. Recovery of DGAT1 from the plant LDs could serve several purposes; recycling of the enzyme as well as removal of a protein that could potentially interfere with the steric hindrance of the oleosins (Tzen et al., 1992). Recycling of DGAT1 is unlikely however, as it would require re-folding after extraction of the large oligomeric form found on the LD. The opportunity for removal from the LD may be afforded before complete disassociation from the ER, or when LDs with incomplete oleosin coatings re-fuse with developing LDs in the ER membrane (Roberts et al., 2008).

Di-arginines also have a role in the assembly of heteromultimeric membrane proteins (Shikano and Li, 2003; Michelsen et al., 2005); however, it is not clear if this is the case for DGAT1. For example, N-terminal truncation prevented dimerization of both human and *Brassica napus* DGAT1s (Zhang et al., 2014; Caldo et al., 2017) whereas similarly treated mouse DGAT1 (McFie et al., 2010) and ZmL (Figure 10) still formed dimers (but not higher order oligomers). Overall, formation of DGAT1 oligomers may assist in stabilizing the protein in the ER and preventing it from dissociating with mature LDs.

## Conclusion

We report on substantial differences in length, charge, and di-arginine motifs between the two forms of DGAT1 in the grasses. It is unclear however, if there is any sub functionalization of the two grass DGAT1s or they are simply an evolutionary feature based on genome duplication and subsequent re-arrangement. Our N-terminal truncated DGAT1s agreed with previous results in showing a role for the N-terminus in oligomerization. The differing performances of the chimeric DGAT1s suggests there is an interaction between the N- and C-termini. Such an interaction has already been postulated to occur, where Caldo et al. (2017) proposed an autoinhibitory sequence of the variable N-terminus. The nature of any potential interaction is currently unknown; as such we are continuing to investigate if the N-terminal di-arginine motifs have a role. In addition, our topological studies suggested the C-terminal maize DGAT1 is in the ER lumen, as the proposed topology of the mammalian orthologs (McFie et al., 2010). A more comprehensive proteomic analysis such as multidimensional protein identification technology would be a valuable approach to investigate the positions of DGAT1 integral membrane residues (Wu et al., 2003; Wang et al., 2012; Lee and Kim, 2014).

## Data availability statement

The nucleotide and peptide sequences presented in this study are deposited in the NCBI GenBank repository with accession numbers shown as follows: Tm (BankIt2601646 BSeq#1 ON959599), Tm(S197A) (BankIt2601652 BSeq#1 ON959601), At (BankIt2601651 BSeq#1 ON959600), OsS (BankIt2601657 BSeq#1 ON959602), OsL (BankIt2601658 BSeq#1 ON959603), ZmS (BankIt2601663 BSeq#1 ON959604), ZmL (BankIt2601666 BSeq#1 ON959605 for *S. cervisiae*, BankIt2601669 BSeq#1 ON959606 for *C. sativa*), ?N Tm (ON946748), ?N At (ON946747), ?N OsS (ON946749), ?N OsL (ON946750), ?N ZmS (ON946751), ?N ZmL (ON946752 for *S. cerevisiae*, ON946753 for *C. sativa*), Tm::ZmS (BankIt2601628 BSeq#1 ON959591 for *S. cerevisiae*, BankIt2601629 BSeq#1 ON959592 for *C. sativa*), Tm::ZmL (BankIt2601630 BSeq#1 ON959593 for *S. cerevisiae*, BankIt2601631 BSeq#1 ON959594 for *C. sativa*), ZmS::Tm (BankIt2601632 BSeq#1 ON959595), ZmL::Tm (BankIt2601634 BSeq#1 ON959596), ZmS::Tm(S170A) (BankIt2601635 BSeq#1 ON959597), ZmL::Tm(S189A) (BankIt2601636 BSeq#1 ON959598), At::ZmL (BankIt2601626 BSeq#1 ON959589), ZmL::At (BankIt2601627 BSeq#1 ON959590), Xp::ZmL-HA151::V5 (BankIt2601037 BSeq#1 ON959580), Xp:: ZmL-HA186::V5 (BankIt2601042 BSeq#1 ON959581), Xp:: ZmL-HA213::V5 (BankIt2601067 BSeq#1 ON959582), Xp:: ZmL-HA251::V5 (BankIt2601070 BSeq#1 ON959583), Xp:: ZmL-HA263::V5 (BankIt2601073 BSeq#1 ON959584), Xp:: ZmL-HA296::V5 (BankIt2601075 BSeq#1 ON959585), Xp:: ZmL-HA338::V5 (BankIt2601079 BSeq#1 ON959586), Xp:: ZmL-HA388::V5 (BankIt2601080 BSeq#1 ON959587), Xp::ZmL-HA473::V5 (BankIt2601081 BSeq#1 ON959588), and Xp::ZmL::V5 (BankIt2600699 BSeq#1 ON959579). The data available at https://apc01.safelinks.protection.outlook.com/?url=https%3A%2F%2Fwww.ncbi.nlm.nih.gov%2FGenbank%2Fupdate.html&data=05%7C01%7Csomrutai.winichayakul%40agresearch.co.nz%7Ccfc6f469d8c74e3a70c808da633b3e7c%7C0dce4a686d804298847ac04815157957%7C0%7C0%7C637931403369601110%7CUnknown%7CTWFpbGZsb3d8eyJWIjoiMC4wLjAwMDAiLCJQIjoiV2luMzIiLCJBTiI6Ik1haWwiLCJXVCI6Mn0%3D%7C3000%7C%7C%7C&sdata=zC5PH1yCwDpJXqtyzrVzOkte0UYo4VwUBxR1eFgV%2BOU%3D&reserved=0.

## Author contributions

NR and RM generated blast searching databases of the DGAT1 sequences and gene analysis. NR, MR, and AC designed the chimeric DGAT1 constructs for yeast expression. MR and AC determined lipid content in yeast experiment. RC and SW characterized the chimeric DGAT1 oligomerization and topology and prepared microsomal protein and immunoblotting. NR and SW designed the chimeric DGAT1 constructs for Camelina expression, designed the project, and wrote the manuscript. SW, HX, and TC performed Camelina transformation, lipid extraction from transgenic Camelina seeds and lipid analysis. NR and GB conceived the project and supervised the research. All authors have read and agreed to the published version of the manuscript.

## Footnotes

1
www.ncbi.nlm.nih.gov/

2
https://www.geneious.com

3
https://alphafold.ebi.ac.uk
