# Supplementary material for: An alternative angiosperm DGAT1 topology and potential motifs in the N-terminus
Source: Front Plant Sci. 2022 Sep 16;13:951389. doi: 10.3389/fpls.2022.951389 (PMC9523541; doi:10.3389/fpls.2022.951389)
Supplement: Supplementary file 1 [file Table_1.pdf]

Supplementary Table 1. Description and peptide sequences of full length, chimeric, ΔN DGAT1, and internally tagged peptide sequences expressed in *Saccharomyces cerevisiae* and *Camelina sativa*.

N-terminal DGAT1 parent	C-terminal DGAT1 parent	N-term TAG	HA TAG POS^{n}	C-terminal TAG	Construct Abbreviation	Expressed in:
Tropaeolum majus	*Tropaeolum majus*	N/A	N/A	V5-6xHis	**Tm**	*S. cerevisiae*
Tropaeolum majus	*Tropaeolum majus* (S197A)	N/A	N/A	V5-6xHis	**Tm(S197A)**	*C. sativa*
Arabidopsis thaliana	*Arabidopsis thaliana*	N/A	N/A	V5-6xHis	**At**	*S. cerevisiae*
Oryza sativa (short)	*Oryza sativa* (short)	N/A	N/A	V5-6xHis	**OsS**	*S. cerevisiae*
Oryza sativa (long)	*Oryza sativa* (long)	N/A	N/A	V5-6xHis	**OsL**	*S. cerevisiae*
Zea mays (short)	*Zea mays* (short)	N/A	N/A	V5-6xHis	**ZmS**	*S. cerevisiae*<br>*C. sativa*
Zea mays (long)	*Zea mays* (long)	N/A	N/A	V5-6xHis	**ZmL**	*S. cerevisiae*<br>*C. sativa*
N-terminus truncated	*Tropaeolum majus*	N/A	N/A	V5-6xHis	**ΔN Tm**	*S. cerevisiae*
N-terminus truncated	*Arabidopsis thaliana*	N/A	N/A	V5-6xHis	**ΔN At**	*S. cerevisiae*
N-terminus truncated	*Oryza sativa* (short)	N/A	N/A	V5-6xHis	**ΔN OsS**	*S. cerevisiae*
N-terminus truncated	*Oryza sativa* (long)	N/A	N/A	V5-6xHis	**ΔN OsL**	*S. cerevisiae*
N-terminus truncated	*Zea mays* (short)	N/A	N/A	V5-6xHis	**ΔN ZmS**	*S. cerevisiae*
N-terminus truncated	*Zea mays* (long)	N/A	N/A	V5-6xHis	**ΔN ZmL**	*S. cerevisiae*<br>*C. sativa*
Tropaeolum majus	*Zea mays* (short)	N/A	N/A	V5-6xHis	**Tm::ZmS**	*S. cerevisiae*<br>*C. sativa*
Tropaeolum majus	*Zea mays* (long)	N/A	N/A	V5-6xHis	**Tm::ZmL**	*S. cerevisiae*<br>*C. sativa*
Zea mays (short)	*Tropaeolum majus*	N/A	N/A	V5-6xHis	**ZmS::Tm**	*S. cerevisiae*
Zea mays (long)	*Tropaeolum majus*	N/A	N/A	V5-6xHis	**ZmL::Tm**	*S. cerevisiae*
Zea mays (short)	*Tropaeolum majus* (S170A)	N/A	N/A	V5-6xHis	**ZmS::Tm(S170A)**	*C. sativa*
Zea mays (long)	*Tropaeolum majus* (S189A)	N/A	N/A	V5-6xHis	**ZmL::Tm(S189A)**	*C. sativa*
Arabidopsis thaliana	*Zea mays* (long)	N/A	N/A	V5-6xHis	**At::ZmL**	*S. cerevisiae*
Zea mays (long)	*Arabidopsis thaliana*	N/A	N/A	V5-6xHis	**ZmL::At**	*S. cerevisiae*
Zea mays (long)	*Zea mays* (long)	Xp	151	V5-6xHis	**Xp::ZmL-HA^{151}::V5**	*S. cerevisiae*
Zea mays (long)	*Zea mays* (long)	Xp	186	V5-6xHis	**Xp:: ZmL-HA^{186}::V5**	*S. cerevisiae*
Zea mays (long)	*Zea mays* (long)	Xp	213	V5-6xHis	**Xp:: ZmL-HA^{213}::V5**	*S. cerevisiae*
Zea mays (long)	*Zea mays* (long)	Xp	251	V5-6xHis	**Xp:: ZmL-HA^{251}::V5**	*S. cerevisiae*

Zea mays (long)	*Zea mays* (long)	Xp	263	V5-6xHis	**Xp:: ZmL-HA^{263}::V5**	*S. cerevisiae*
Zea mays (long)	*Zea mays* (long)	Xp	296	V5-6xHis	**Xp:: ZmL-HA^{296}::V5**	*S. cerevisiae*
Zea mays (long)	*Zea mays* (long)	Xp	338	V5-6xHis	**Xp:: ZmL-HA^{338}::V5**	*S. cerevisiae*
Zea mays (long)	*Zea mays* (long)	Xp	388	V5-6xHis	**Xp:: ZmL-HA^{388}::V5**	*S. cerevisiae*
Zea mays (long)	*Zea mays* (long)	Xp	473	V5-6xHis	**Xp:: ZmL-HA^{473}::V5**	*S. cerevisiae*
Zea mays (long)	*Zea mays* (long)	Xp	N/A	V5-6xHis	**Xp::ZmL::V5**	*S. cerevisiae*

Tm (*ON959599*)
MAVAESSQNTTTMSGHGDSDLNNFRRRKPSSSVIEPSSSGFTSTNGVPATGHVAENRDQDRVGAMENATGSVNL
IGNGGGVVIGNEEKQVGETDIRFTYRPSFPAHRRVRESPLSSDAIFKQSHAGLFNLCIVVLIAVNSRLIIENLM
KYGWLIDTGFWFSSRSLGDWSIFMCCLTLPIFPLAAFIVEKLVQRNHISELVAVLLHVIVSTAAVLYPVIVILT
CDSVYMSGVVLMLFGCIMWLKLVSYAHTSSDIRTLAKSGYKGDAHPNSTIVSCSYDVSLKSLAYFMVAPTLCYQ
PSYPRSSCIRKGWVVRQFVKLIVFIGLMGFIIEQYINPIVRNSKHPLKGDFLYAIERVLKLSVPNLYVWLCMFY
SFFHLWLNILAELLRFGDREFYKDWWNAKTVAEYWKMWNMPVHRWMVRHLYFPCLRNGIPKEGAIIIAFLVSGA
FHELCIAVPCHVFKLWAFIGIMFQVPLVLITNYLQEKFSNSMVGNMIFWFIFCILGQPMCVLLYYHDLINLKEK
AKGELRGHPFEGKPIPNPLLGLDSTRTGHHHHHH

Tm(S197A) (*ON959601*)
MAVAESSQNTTTMSGHGDSDLNNFRRRKPSSSVIEPSSSGFTSTNGVPATGHVAENRDQDRVGAMENATGSVNL
IGNGGGVVIGNEEKQVGETDIRFTYRPSFPAHRRVRESPLSSDAIFKQSHAGLFNLCIVVLIAVNSRLIIENLM
KYGWLIDTGFWFSSRSLGDWSIFMCCLTLPIFPLAAFIVEKLVQRNHIAELVAVLLHVIVSTAAVLYPVIVILT
CDSVYMSGVVLMLFGCIMWLKLVSYAHTSSDIRTLAKSGYKGDAHPNSTIVSCSYDVSLKSLAYFMVAPTLCYQ
PSYPRSSCIRKGWVVRQFVKLIVFIGLMGFIIEQYINPIVRNSKHPLKGDFLYAIERVLKLSVPNLYVWLCMFY
SFFHLWLNILAELLRFGDREFYKDWWNAKTVAEYWKMWNMPVHRWMVRHLYFPCLRNGIPKEGAIIIAFLVSGA
FHELCIAVPCHVFKLWAFIGIMFQVPLVLITNYLQEKFSNSMVGNMIFWFIFCILGQPMCVLLYYHDLINLKEK
AKGELRGHPFEGKPIPNPLLGLDSTRTGHHHHHH

At (*ON959600*)
MAILDSAGVTTVTENGGGEFVDLDRLRRRKSRSDSSNGLLLSGSDNNSPSDDVGAPADVRDRIDSVVNDDAQGT
ANLAGDNNGGGDNNGGGRGGGEGRGNADATFTYRPSVPAHRRARESPLSSDAIFKQSHAGLFNLCVVVLIAVNS
RLIIENLMKYGWLIRTDFWFSSRSLRDWPLFMCCISLSIFPLAAFTVEKLVLQKYISEPVVIFLHIIITMTEVL
YPVYVTLRCDSAFLSGVTLMLLTCIVWLKLVSYAHTSYDIRSLANAADKANPEVSYYVSLKSLAYFMVAPTLCY
QPSYPRSACIRKGWVARQFAKLVIFTGFMGFIIEQYINPIVRNSKHPLKGDLLYAIERVLKLSVPNLYVWLCMF
YCFFHLWLNILAELLCFGDREFYKDWWNAKSVGDYWRMWNMPVHKWMVRHIYFPCLRSKIPKTLAIIIAFLVSA
VFHELCIAVPCRLFKLWAFLGIMFQVPLVFITNYLQERFGSTVGNMIFWFIFCIFGQPMCVLLYYHDLMNRKGS
MS**AKGELRGHPFEGKPIPNPLLGLDSTRTGHHHHHH**

OsS (*ON959602*)
MAPPPSLAPDRGGGEPDDALRLRARAAAAAGDAPAPQQQQEQRHQEQQQQLLWYRASAPAHRRVRESPLSSDAI
FRQSHAGLLNLCIVVLVAVNSRLIIENLMKYGLLIRAGFWFSGTSLADWPLLMCCLTLPTFPLAALMVEKLAQR
KLISKHVVILLHIVITTSVLVYPVVVILKCDSAVLSGFVLMFLASIIWLKLVSFAHTNYDIRMLSKSIEKGVTH
DISIDPENIKWPTFKRLSYFMLAPTLCYQPSYPRTTYIRKGWVVRQLIKCLVFTGLMGFIIEQYINPIVKNSKH
PLKGNFLNAIERVLKLSVPTLYVWLCMFYCFFHLWLNILAELLCFGDREFYKDWWNAKTVEEYWRMWNMPVHKW
VIRHIYFPCIRNGFSKGVAILISFLVSAAFHELCVAVPCHIFKFWAFIGIMFQIPLVFLTKYLQDKFNNTMVGN
MIFWFFFSILGQPMCVLLYYHDVMNRQQAQTNR**AKGELRGHPFEGKPIPNPLLGLDSTRTGHHHHHH**

OsL (*ON959603*)
MVGSDGDGDGGGGEAHAPAAPAHHHRRPPRPRGGSGAIVEGFAAALRRRIRSGAAAAARASFGGDSGDEAASGE
PSSSSSSSPSRRRGGDSNGAEASSAAGGGGGRGGGGDFSAFTFRAAAPVHRKAKESPLSSDAIFKQSHAGLFNL
CIVVLVAVNSRLIIENLMKYGLLIRAGFWFNDKSLRDWPLLMCCLSLPAFPLGAFAVEKLAFNNVITDAVATCL
HIFLSTTEIVYPVLVILKCDSAVLSGFLLIFIACIVWLKLVSFAHTNHDIRQLTMGGKKVDNELSTVDMDNLQP
PTLGNLIYFMMAPTLCYQPSYPRTSCVRKGWLIRQIILYLIFTGLQGFIIEQYINPIVVNSQHPLKGGLLNAVE
TVLKLSLPNVYLWLCMFYAFFHLWLSILAEILRFGDREFYKDWWNAKTIDEYWRKWNMPVHKWVVRHIYFPCMR

NGISKEVAVLISFLVSAVLHEICVAVPCRILKFWAFLGIMLQIPLIVLTAYLKSKFRDTMVGNMIFWFFFCIYG<br>QPMCLLLYYHDVMNRIEKAR**AKGELRGHPFEGKPIPNPLLGLDSTRTGHHHHHH**
**ZmS** (*ON959604*)<br>MAPPPSMPAASDRAGPGRDAGDSSSLRLRRAPSADAGDLAGDSSGGLRENGEPQSPTNPPPQEQQQHEMLYYRA<br>SAPAHRRVKESPLSSDAIFRQSHAGLLNLCIVVLIAVNSRLIIENLMKYGLLIRAGFWFSARSLGDWPLLMCCL<br>TLPVFPLVALMAEKLITRKLIGEHVVILLHIIITTSAIVYPVVVTLKCDSAVLSGFVLMFLASIMWMKLVSYAH<br>TNYDIRVLSKSTEKGAAYGNYVDPENMKDPTFKSLVYFMLAPTLCYQPTYPQTTCIRKGWVTQQLIKCVVFTGL<br>MGFIIEQYINPIVKNSKHPLKGNFLNAIERVLKLSVPTLYVWLCMFYCFFHLWLNIVAELLCFGDREFYKDWWN<br>AKTVEEYWRMWNMPVHKWIIRHIYFPCIRKGFSRGVAILISFLVSAVFHEICIAVPCHIFKFWAFSGIMFQIPL<br>VFLTRYLHATFKHVMVGNMIFWFFFSIVGQPMCVLLYYHDVMNRQAQASR**AKGELRGHPFEGKPIPNPLLGLDS**<br>**TRTGHHHHHH**
**ZmL** (*ON959605* for *S. cerevisiae*, *ON959606* for *C. sativa*)<br>MADSEDAPPAVHRRPPRPARGAAAAQGFAAALRRRLRSGAAVAARASFAADSGDESGPGEPSSSRRRDNSGGAS<br>SAAGGRAGAGDFSAFTFRAAAPVHRKAKESPLSSDAIFKQSHAGLFNLCIVVLVAVNSRLIIENLMKYGLLIRS<br>GFWFNATSLRDWPLLMCCLSLPIFPLGAFAVEKLAFNNLISDPATTCFHILFTTFEIVYPVLVILKCDSAVLSG<br>FVLMFIACIVWLKLVSFAHTNHDIRKLITSGKKVDNELTAAGIDNLQAPTLGSLTYFMMAPTLCYQPSYPRTPY<br>VRKGWLVRQVILYLIFTGLQGFIIEQYINPIVVNSQHPLMGGLLNAVETVLKLSLPNVYLWLCMFYCLFHLWLN<br>ILAEILRFGDREFYKDWWNAKTIDEYWRKWNMPVHKWIVRHIYFPCMRNGISKEVAVFISFFVSAVLHELCVAV<br>PCHILKFWAFLGIMLQIPLIILTSYLKNKFSDTMVGNMIFWFFFCIYGQPMCVLLYYHDVMNRTEKAK**AKGELR**<br>**GHPFEGKPIPNPLLGLDSTRTGHHHHHH**
**ΔN Tm** (*ON946748*)<br>**MGGGS**IRFTYRPSFPAHRRVRESPLSSDAIFKQSHAGLFNLCIVVLIAVNSRLIIENLMKYGWLIDTGFWFSSR<br>SLGDWSIFMCCLTLPIFPLAAFIVEKLVQRNHISELVAVLLHVIVSTAAVLYPVIVILTCDSVYMSGVVLMLFG<br>CIMWLKLVSYAHTSSDIRTLAKSGYKGDAHPNSTIVSCSYDVSLKSLAYFMVAPTLCYQPSYPRSSCIRKGWVV<br>RQFVKLIVFIGLMGFIIEQYINPIVRNSKHPLKGDFLYAIERVLKLSVPNLYVWLCMFYSFFHLWLNILAELLR<br>FGDREFYKDWWNAKTVAEYWKMWNMPVHRWMVRHLYFPCLRNGIPKEGAIIIAFLVSGAFHELCIAVPCHVFKL<br>WAFIGIMFQVPLVLITNYLQEKFSNSMVGNMIFWFIFCILGQPMCVLLYYHDLINLKEK**AKGELRGHPFEGKPI**<br>**PNPLLGLDSTRTGHHHHHH**
**ΔN At** (*ON946747*)<br>**MGGGS**ATFTYRPSVPAHRRARESPLSSDAIFKQSHAGLFNLCVVVLIAVNSRLIIENLMKYGWLIRTDFWFSSR<br>SLRDWPLFMCCISLSIFPLAAFTVEKLVLQKYISEPVVIFLHIIITMTEVLYPVYVTLRCDSAFLSGVTLMLLT<br>CIVWLKLVSYAHTSYDIRSLANAADKANPEVSYYVSLKSLAYFMVAPTLCYQPSYPRSACIRKGWVARQFAKLV<br>IFTGFMGFIIEQYINPIVRNSKHPLKGDLLYAIERVLKLSVPNLYVWLCMFYCFFHLWLNILAELLCFGDREFY<br>KDWWNAKSVGDYWRMWNMPVHKWMVRHIYFPCLRSKIPKTLAIIIAFLVSAVFHELCIAVPCRLFKLWAFLGIM<br>FQVPLVFITNYLQERFGSTVGNMIFWFIFCIFGQPMCVLLYYHDLMNRKGSMS**AKGELRGHPFEGKPIPNPLLG**<br>**LDSTRTGHHHHHH**
**ΔN OsS** (*ON946749*)<br>**MGGGS**QLLWYRASAPAHRRVRESPLSSDAIFRQSHAGLLNLCIVVLVAVNSRLIIENLMKYGLLIRAGFWFSGT<br>SLADWPLLMCCLTLPTFPLAALMVEKLAQRKLISKHVVILLHIVITTSVLVYPVVVILKCDSAVLSGFVLMFLA<br>SIIWLKLVSFAHTNYDIRMLSKSIEKGVTHDISIDPENIKWPTFKRLSYFMLAPTLCYQPSYPRTTYIRKGWVV<br>RQLIKCLVFTGLMGFIIEQYINPIVKNSKHPLKGNFLNAIERVLKLSVPTLYVWLCMFYCFFHLWLNILAELLC<br>FGDREFYKDWWNAKTVEEYWRMWNMPVHKWVIRHIYFPCIRNGFSKGVAILISFLVSAAFHELCVAVPCHIFKF<br>WAFIGIMFQIPLVFLTKYLQDKFNNTMVGNMIFWFFFSILGQPMCVLLYYHDVMNRQQAQTNR**AKGELRGHPFE**<br>**GKPIPNPLLGLDSTRTGHHHHHH**
**ΔN OsL** (*ON946750*)<br>**MGGGS**SAFTFRAAAPVHRKAKESPLSSDAIFKQSHAGLFNLCIVVLVAVNSRLIIENLMKYGLLIRAGFWFNDK<br>SLRDWPLLMCCLSLPAFPLGAFAVEKLAFNNVITDAVATCLHIFLSTTEIVYPVLVILKCDSAVLSGFLLIFIA<br>CIVWLKLVSFAHTNHDIRQLTMGGKKVDNELSTVDMDNLQPPTLGNLIYFMMAPTLCYQPSYPRTSCVRKGWLI<br>RQIILYLIFTGLQGFIIEQYINPIVVNSQHPLKGGLLNAVETVLKLSLPNVYLWLCMFYAFFHLWLSILAEILR<br>FGDREFYKDWWNAKTIDEYWRKWNMPVHKWVVRHIYFPCMRNGISKEVAVLISFLVSAVLHEICVAVPCRILKF<br>WAFLGIMLQIPLIVLTAYLKSKFRDTMVGNMIFWFFFCIYGQPMCLLLYYHDVMNRIEKAR**AKGELRGHPFEGK**<br>**PIPNPLLGLDSTRTGHHHHHH**
**ΔN ZmS** (*ON946751*)<br>**MGGGS**EMLYYRASAPAHRRVKESPLSSDAIFRQSHAGLLNLCIVVLIAVNSRLIIENLMKYGLLIRAGFWFSAR<br>SLGDWPLLMCCLTLPVFPLVALMAEKLITRKLIGEHVVILLHIIITTSAIVYPVVVTLKCDSAVLSGFVLMFLA

SIMWMKLVSYAHTNYDIRVLSKSTEKGAAYGNYVDPENMKDPTFKSLVYFMLAPTLCYQPTYPQTTCIRKGWVT<br>QQLIKCVVFTGLMGFIIEQYINPIVKNSKHPLKGNFLNAIERVLKLSVPTLYVWLCMFYCFFHLWLNIVAELLC<br>FGDREFYKDWWNAKTVEEYWRMWNMPVHKWIIRHIYFPCIRKGFSRGVAILISFLVSAVFHEICIAVPCHIFKF<br>WAFSGIMFQIPLVFLTRYLHATFKHVMVGNMIFWFFFSIVGQPMCVLLYYHDVMNRQAQASR**AKGELRGHPFEG<br>KPIPNPLLGLDSTRTGHHHHHH**
**ΔN ZmL** (*ON946752* for *S. cerevisiae*, *ON946753* for *C. sativa*)<br>**MGGGS**SAFTFRAAAPVHRKAKESPLSSDAIFKQSHAGLFNLCIVVLAVNSRLIIENLMKYGLLIRSGFWFNAT<br>SLRDWPLLMCCLSLPIFPLGAFAVEKLAFNNLISDPATTCFHILFTTFEIVYPVLVILKCDSAVLSGFVLMFIA<br>CIVWLKLVSFAHTNHDIRKLITSGKKVDNELTAAGIDNLQAPTLGSLTYFMMAPTLCYQPSYPRTPYVRKGWLV<br>RQVILYLIFTGLQGFIIEQYINPIVVNSQHPLMGGLLNAVETVLKLSLPNVYLWLCMFYCLFHLWLNILAEILR<br>FGDREFYKDWWNAKTIDEYWRKWNMPVHKWIVRHIYFPCMRNGISKEVAVFISFFVSAVLHELCVAVPCHILKF<br>WAFLGIMLQIPLIILTSYLKNKFSDTMVGNMIFWFFFCIYGQPMCVLLYYHDVMNRTEKAK**AKGELRGHPFEGK<br>PIPNPLLGLDSTRTGHHHHHH**
**Tm::ZmS** (*ON959591* for *S. cerevisiae*, *ON959592* for *C. sativa*)<br>MAVAESSQNTTTMSGHGDSDLNNFRRRKPSSSVIEPSSSGFTSTNGVPATGHVAENRDQDRVGAMENATGSVNL<br>IGNGGGVVIGNEEKQVGETDIRFTYRPSFPAHRRVRESPLSSDAIFRQSHAGLLNLCIVVLIAVNSRLIIENLM<br>KYGLLIRAGFWFSARSLGDWPLLMCCLTLPVFPLVALMAEKLITRKLIGEHVVILLHIIITTSAIVYPVVVTLK<br>CDSAVLSGFVLMFLASIMWMKLVSYAHTNYDIRVLSKSTEKGAAYGNYVDPENMKDPTFKSLVYFMLAPTLCYQ<br>PTYPQTTCIRKGWVTQQLIKCVVFTGLMGFIIEQYINPIVKNSKHPLKGNFLNAIERVLKLSVPTLYVWLCMFY<br>CFFHLWLNIVAELLCFGDREFYKDWWNAKTVEEYWRMWNMPVHKWIIRHIYFPCIRKGFSRGVAILISFLVSAV<br>FHEICIAVPCHIFKFWAFSGIMFQIPLVFLTRYLHATFKHVMVGNMIFWFFFSIVGQPMCVLLYYHDVMNRQAQ<br>ASR**AKGELRGHPFEGKPIPNPLLGLDSTRTGHHHHHH**
**Tm::ZmL** (*ON959593* for *S. cerevisiae*, *ON959594* for *C. sativa*)<br>MAVAESSQNTTTMSGHGDSDLNNFRRRKPSSSVIEPSSSGFTSTNGVPATGHVAENRDQDRVGAMENATGSVNL<br>IGNGGGVVIGNEEKQVGETDIRFTYRPSFPAHRRVRESPLSSDAIFKQSHAGLFNLCIVVLVAVNSRLIIENLM<br>KYGLLIRSGFWFNATSLRDWPLLMCCLSLPIFPLGAFAVEKLAFNNLISDPATTCFHILFTTFEIVYPVLVILK<br>CDSAVLSGFVLMFIACIVWLKLVSFAHTNHDIRKLITSGKKVDNELTAAGIDNLQAPTLGSLTYFMMAPTLCYQ<br>PSYPRTPYVRKGWLVRQVILYLIFTGLQGFIIEQYINPIVVNSQHPLMGGLLNAVETVLKLSLPNVYLWLCMFY<br>CLFHLWLNILAEILRFGDREFYKDWWNAKTIDEYWRKWNMPVHKWIVRHIYFPCMRNGISKEVAVFISFFVSAV<br>LHELCVAVPCHILKFWAFLGIMLQIPLIILTSYLKNKFSDTMVGNMIFWFFFCIYGQPMCVLLYYHDVMNRTEK<br>AK**AKGELRGHPFEGKPIPNPLLGLDSTRTGHHHHHH**
**ZmS::Tm** (*ON959595***)**<br>MAPPPSMPAASDRAGPGRDAGDSSSLRLRRAPSADAGDLAGDSSGGLRENGEPQSPTNPPPQEQQQHEMLYYRA<br>SAPAHRRVKESPLSSDAIFKQSHAGLFNLCIVVLIAVNSRLIIENLMKYGWLIDTGFWFSSRSLGDWSIFMCCL<br>TLPIFPLAAFIVEKLVQRNHISELVAVLLHVIVSTAAVLYPVIVILTCDSVYMSGVVLMLFGCIMWLKLVSYAH<br>TSSDIRTLAKSGYKGDAHPNSTIVSCSYDVSLKSLAYFMVAPTLCYQPSYPRSSCIRKGWVVRQFVKLIVFIGL<br>MGFIIEQYINPIVRNSKHPLKGDFLYAIERVLKLSVPNLYVWLCMFYSFFHLWLNILAELLRFGDREFYKDWWN<br>AKTVAEYWKMWNMPVHRWMVRHLYFPCLRNGIPKEGAIIIAFLVSGAFHELCIAVPCHVFKLWAFIGIMFQVPL<br>VLITNYLQEKFSNSMVGNMIFWFIFCILGQPMCVLLYYHDLINLKEK**AKGELRGHPFEGKPIPNPLLGLDSTRT<br>GHHHHHH**
**ZmL::Tm** (*ON959596*)<br>MADSEDAPPAVHRRPPRPARGAAAAQGFAAALRRRLRSGAAVAARASFAADSGDESGPGEPSSSRRRDNSGGAS<br>SAAGGRAGAGDFSAFTFRAAAPVHRKAKESPLSSDAIFKQSHAGLFNLCIVVLIAVNSRLIIENLMKYGWLIDT<br>GFWFSSRSLGDWSIFMCCLTLPIFPLAAFIVEKLVQRNHISELVAVLLHVIVSTAAVLYPVIVILTCDSVYMSG<br>VVLMLFGCIMWLKLVSYAHTSSDIRTLAKSGYKGDAHPNSTIVSCSYDVSLKSLAYFMVAPTLCYQPSYPRSSC<br>IRKGWVVRQFVKLIVFIGLMGFIIEQYINPIVRNSKHPLKGDFLYAIERVLKLSVPNLYVWLCMFYSFFHLWLN<br>ILAELLRFGDREFYKDWWNAKTVAEYWKMWNMPVHRWMVRHLYFPCLRNGIPKEGAIIIAFLVSGAFHELCIAV<br>PCHVFKLWAFIGIMFQVPLVLITNYLQEKFSNSMVGNMIFWFIFCILGQPMCVLLYYHDLINLKEK**AKGELRGH<br>PFEGKPIPNPLLGLDSTRTGHHHHHH**
**ZmS::Tm(S170A)**(*ON959597*)<br>MAPPPSMPAASDRAGPGRDAGDSSSLRLRRAPSADAGDLAGDSSGGLRENGEPQSPTNPPPQEQQQHEMLYYRA<br>SAPAHRRVKESPLSSDAIFKQSHAGLFNLCIVVLIAVNSRLIIENLMKYGWLIDTGFWFSSRSLGDWSIFMCCL<br>TLPIFPLAAFIVEKLVQRNHIAELVAVLLHVIVSTAAVLYPVIVILTCDSVYMSGVVLMLFGCIMWLKLVSYAH<br>TSSDIRTLAKSGYKGDAHPNSTIVSCSYDVSLKSLAYFMVAPTLCYQPSYPRSSCIRKGWVVRQFVKLIVFIGL<br>MGFIIEQYINPIVRNSKHPLKGDFLYAIERVLKLSVPNLYVWLCMFYSFFHLWLNILAELLRFGDREFYKDWWN

AKTVAEYWKMWNMPVHRWMVRHLYFPCLRNGIPKEGAIIIAFLVSGAFHELCIAVPCHVFKLWAFIGIMFQVPL
VLITNYLQEKFSNSMVGNMIFWFIFCILGQPMCVLLYYHDLINLKEK**AKGELRGHPFEGKPIPNPLLGLDSTRT
GHHHHHH**

ZmL::Tm(S189A)(*ON959598*)
MADSEDAPPAVHRRPPRPARGAAAAQGFAAALRRRLRSGAAVAARASFAADSGDESGPGEPSSSRRRDNSGGAS
SAAGGRAGAGDFSAFTFRAAAPVHRKAKESPLSSDAIFKQSHAGLFNLCIVVLIAVNSRLIIENLMKYGWLIDT
GFWFSSRSLGDWSIFMCCLTLPIFPLAAFIVEKLVQRNHIAELVAVLLHVIVSTAAVLYPVIVILTCDSVYMSG
VVLMLFGCIMWLKLVSYAHTSSDIRTLAKSGYKGDAHPNSTIVSCSYDVSLKSLAYFMVAPTLCYQPSYPRSSC
IRKGWVVRQFVKLIVFIGLMGFIIEQYINPIVRNSKHPLKGDFLYAIERVLKLSVPNLYVWLCMFYSFFHLWLN
ILAELLRFGDREFYKDWWNAKTVAEYWKMWNMPVHRWMVRHLYFPCLRNGIPKEGAIIIAFLVSGAFHELCIAV
PCHVFKLWAFIGIMFQVPLVLITNYLQEKFSNSMVGNMIFWFIFCILGQPMCVLLYYHDLINLKEK**AKGELRGH
PFEGKPIPNPLLGLDSTRTGHHHHHH**

At::ZmL (*ON959589*)
MAILDSAGVTTVTENGGGEFVDLDRLRRRKSRSDSSNGLLLSGSDNNSPSDDVGAPADVRDRIDSVVNDDAQGT
ANLAGDNNGGGDNNGGGRGGGEGRGNADATFTYRPSVPAHRRARESPLSSDAIFKQSHAGLFNLCIVVLVAVNS
RLIIENLMKYGLLIRSGFWFNATSLRDWPLLMCCLSLPIFPLGAFAVEKLAFNNLISDPATTCFHILFTTFEIV
YPVLVILKCDSAVLSGFVLMFIACIVWLKLVSFAHTNHDIRKLITSGKKVDNELTAAGIDNLQAPTLGSLTYFM
MAPTLCYQPSYPRTPYVRKGWLVRQVILYLIFTGLQGFIIEQYINPIVVNSQHPLMGGLLNAVETVLKLSLPNV
YLWLCMFYCLFHLWLNILAEILRFGDREFYKDWWNAKTIDEYWRKWNMPVHKWIVRHIYFPCMRNGISKEVAVF
ISFFVSAVLHELCVAVPCHILKFWAFLGIMLQIPLIILTSYLKNKFSDTMVGNMIFWFFFCIYGQPMCVLLYYH
DVMNRTEKAK**AKGELRGHPFEGKPIPNPLLGLDSTRTGHHHHHH**

ZmL::At (*ON959590*)
MADSEDAPPAVHRRPPRPARGAAAAQGFAAALRRRLRSGAAVAARASFAADSGDESGPGEPSSSRRRDNSGGAS
SAAGGRAGAGDFSAFTFRAAAPVHRKAKESPLSSDAIFKQSHAGLFNLCVVVLIAVNSRLIIENLMKYGWLIRT
DFWFSSRSLRDWPLFMCCISLSIFPLAAFTVEKLVLQKYISEPVVIFLHIIITMTEVLYPVYVTLRCDSAFLSG
VTLMLLTCIVWLKLVSYAHTSYDIRSLANAADKANPEVSYYVSLKSLAYFMVAPTLCYQPSYPRSACIRKGWVA
RQFAKLVIFTGFMGFIIEQYINPIVRNSKHPLKGDLLYAIERVLKLSVPNLYVWLCMFYCFFHLWLNILAELLC
FGDREFYKDWWNAKSVGDYWRMWNMPVHKWMVRHIYFPCLRSKIPKTLAIIIAFLVSAVFHELCIAVPCRLFKL
WAFLGIMFQVPLVFITNYLQERFGSTVGNMIFWFIFCIFGQPMCVLLYYHDLMNRKGSMS**AKGELRGHPFEGKP
IPNPLLGLDSTRTGHHHHHH**

Xp::ZmL-HA^{151}::V5 (*ON959580*)
MGGSHHHHHHGMASMTGGQQMGRDLYDDDDKVPRIQCGGIRRQKMADSEDAPPAVHRRPPRPARGAAAAQGFAA
ALRRRLRSGAAVAARASFAADSGDESGPGEPSSSRRRDNSGGASSAAGGRAGAGDFSAFTFRAAAPVHRKAKES
PLSSDAIFKQSHAGLFNLCIVVLVAVNSRLIIENLMKYGLLIRSGFGSYPYDVPDYAGSWFNATSLRDWPLLMC
CLSLPIFPLGAFAVEKLAFNNLISDPATTCFHILFTTFEIVYPVLVILKCDSAVLSGFVLMFIACIVWLKLVSF
AHTNHDIRK**LITSGKKVDNELTAAGIDNLQAPTLGSLTYFMMAPTLCYQPSYPRTP**YVRKGWLVRQVILYLIFT
GLQGFIIEQYINPIVVNSQHPLMGGLLNAVETVLKLSLPNVYLWLCMFYCLFHLWLNILAEILRFGDREFYKDW
WNAKTIDEYWRKWNMPVHKWIVRHIYFPCMRNGISKEVAVFISFFVSAVLHELCVAVPCHILKFWAFLGIMLQI
PLIILTSYLKNKFSDTMVGNMIFWFFFCIYGQPMCVLLYYHDVMNRTEKAK**GELRGHPFEGKPIPNPLLGLDST
RTGHHHHHH**

Xp:: ZmL-HA^{186}::V5 (*ON959581*)
MGGSHHHHHHGMASMTGGQQMGRDLYDDDDKVPRIQCGGIRRQKMADSEDAPPAVHRRPPRPARGAAAAQGFAA
ALRRRLRSGAAVAARASFAADSGDESGPGEPSSSRRRDNSGGASSAAGGRAGAGDFSAFTFRAAAPVHRKAKES
PLSSDAIFKQSHAGLFNLCIVVLVAVNSRLIIENLMKYGLLIRSGFWFNATSLRDWPLLMCCLSLPIFPLGAFA
VEKLAFNGSYPYDVPDYAGSNLISDPATTCFHILFTTFEIVYPVLVILKCDSAVLSGFVLMFIACIVWLKLVSF
AHTNHDIRK**LITSGKKVDNELTAAGIDNLQAPTLGSLTYFMMAPTLCYQPSYPRTP**YVRKGWLVRQVILYLIFT
GLQGFIIEQYINPIVVNSQHPLMGGLLNAVETVLKLSLPNVYLWLCMFYCLFHLWLNILAEILRFGDREFYKDW
WNAKTIDEYWRKWNMPVHKWIVRHIYFPCMRNGISKEVAVFISFFVSAVLHELCVAVPCHILKFWAFLGIMLQI
PLIILTSYLKNKFSDTMVGNMIFWFFFCIYGQPMCVLLYYHDVMNRTEKAK**GELRGHPFEGKPIPNPLLGLDST
RTGHHHHHH**

Xp:: ZmL-HA^{213}::V5 (*ON959582*)
MGGSHHHHHHGMASMTGGQQMGRDLYDDDDKVPRIQCGGIRRQKMADSEDAPPAVHRRPPRPARGAAAAQGFAA
ALRRRLRSGAAVAARASFAADSGDESGPGEPSSSRRRDNSGGASSAAGGRAGAGDFSAFTFRAAAPVHRKAKES
PLSSDAIFKQSHAGLFNLCIVVLVAVNSRLIIENLMKYGLLIRSGFWFNATSLRDWPLLMCCLSLPIFPLGAFA
VEKLAFNNLISDPATTCFHILFTTFEIVYPVLVIGSYPYDVPDYAGSLKCDSAVLSGFVLMFIACIVWLKLVSF

AHTNHDIRK**LITSGKKVDNELTAAGIDNLQAPTLGSLTYFMMAPTLCYQPSYPRTP**YVRKGWLVRQVILYLIFT
GLQGFIIEQYINPIVVNSQHPLMGGLLNAVETVLKLSLPNVYLWLCMFYCLFHLWLNILAEILRFGDREFYKDW
WNAKTIDEYWRKWNMPVHKWIVRHIYFPCMRNGISKEVAVFISFFVSAVLHELCVAVPCHILKFWAFLGIMLQI
PLIILTSYLKNKFSDTMVGNMIFWFFFCIYGQPMCVLLYYHDVMNRTEKAK**GELRGHPFEGKPIPNPLLGLDST**
RTGHHHHHH

Xp:: ZmL-HA^{251}::V5 (*ON959583*)
MGGSHHHHHHGMASMTGGQQMGRDLYDDDDKVPRIQCGGIRRQKMADSEDAPPAVHRRPPRPARGAAAAQGFAA
ALRRRLRSGAAVAARASFAADSGDESGPGEPSSSRRRDNSGGASSAAGGRAGAGDFSAFTFRAAAPVHRKAKES
PLSSDAIFKQSHAGLFNLCIVVLVAVNSRLIIENLMKYGLLIRSGFWFNATSLRDWPLLMCCLSLPIFPLGAFA
VEKLAFNNLISDPATTCFHILFTTFEIVYPVLVILKCDSAVLSGFVLMFIACIVWLKLVSFAHTNHDIRK**LI**GS
YPYDVPDYAGS**TSGKKVDNELTAAGIDNLQAPTLGSLTYFMMAPTLCYQPSYPRTP**YVRKGWLVRQVILYLIFT
GLQGFIIEQYINPIVVNSQHPLMGGLLNAVETVLKLSLPNVYLWLCMFYCLFHLWLNILAEILRFGDREFYKDW
WNAKTIDEYWRKWNMPVHKWIVRHIYFPCMRNGISKEVAVFISFFVSAVLHELCVAVPCHILKFWAFLGIMLQI
PLIILTSYLKNKFSDTMVGNMIFWFFFCIYGQPMCVLLYYHDVMNRTEKAK**GELRGHPFEGKPIPNPLLGLDST**
RTGHHHHHH

Xp:: ZmL-HA^{263}::V5 (*ON959584*)
MGGSHHHHHHGMASMTGGQQMGRDLYDDDDKVPRIQCGGIRRQKMADSEDAPPAVHRRPPRPARGAAAAQGFAA
ALRRRLRSGAAVAARASFAADSGDESGPGEPSSSRRRDNSGGASSAAGGRAGAGDFSAFTFRAAAPVHRKAKES
PLSSDAIFKQSHAGLFNLCIVVLVAVNSRLIIENLMKYGLLIRSGFWFNATSLRDWPLLMCCLSLPIFPLGAFA
VEKLAFNNLISDPATTCFHILFTTFEIVYPVLVILKCDSAVLSGFVLMFIACIVWLKLVSFAHTNHDIRK**LITS**
GKKVDNELTAGSYPYDVPDYAGS**AGIDNLQAPTLGSLTYFMMAPTLCYQPSYPRTP**YVRKGWLVRQVILYLIFT
GLQGFIIEQYINPIVVNSQHPLMGGLLNAVETVLKLSLPNVYLWLCMFYCLFHLWLNILAEILRFGDREFYKDW
WNAKTIDEYWRKWNMPVHKWIVRHIYFPCMRNGISKEVAVFISFFVSAVLHELCVAVPCHILKFWAFLGIMLQI
PLIILTSYLKNKFSDTMVGNMIFWFFFCIYGQPMCVLLYYHDVMNRTEKAK**GELRGHPFEGKPIPNPLLGLDST**
RTGHHHHHH

Xp:: ZmL-HA^{296}::V5 (*ON959585*)
MGGSHHHHHHGMASMTGGQQMGRDLYDDDDKVPRIQCGGIRRQKMADSEDAPPAVHRRPPRPARGAAAAQGFAA
ALRRRLRSGAAVAARASFAADSGDESGPGEPSSSRRRDNSGGASSAAGGRAGAGDFSAFTFRAAAPVHRKAKES
PLSSDAIFKQSHAGLFNLCIVVLVAVNSRLIIENLMKYGLLIRSGFWFNATSLRDWPLLMCCLSLPIFPLGAFA
VEKLAFNNLISDPATTCFHILFTTFEIVYPVLVILKCDSAVLSGFVLMFIACIVWLKLVSFAHTNHDIRK**LITS**
GKKVDNELTAAGIDNLQAPTLGSLTYFMMAPTLCYQPSYPRTPGSYPYDVPDYAGSYVRKGWLVRQVILYLIFT
GLQGFIIEQYINPIVVNSQHPLMGGLLNAVETVLKLSLPNVYLWLCMFYCLFHLWLNILAEILRFGDREFYKDW
WNAKTIDEYWRKWNMPVHKWIVRHIYFPCMRNGISKEVAVFISFFVSAVLHELCVAVPCHILKFWAFLGIMLQI
PLIILTSYLKNKFSDTMVGNMIFWFFFCIYGQPMCVLLYYHDVMNRTEKAK**GELRGHPFEGKPIPNPLLGLDST**
RTGHHHHHH

Xp:: ZmL-HA^{338}::V5 (*ON959586*)
MGGSHHHHHHGMASMTGGQQMGRDLYDDDDKVPRIQCGGIRRQKMADSEDAPPAVHRRPPRPARGAAAAQGFAA
ALRRRLRSGAAVAARASFAADSGDESGPGEPSSSRRRDNSGGASSAAGGRAGAGDFSAFTFRAAAPVHRKAKES
PLSSDAIFKQSHAGLFNLCIVVLVAVNSRLIIENLMKYGLLIRSGFWFNATSLRDWPLLMCCLSLPIFPLGAFA
VEKLAFNNLISDPATTCFHILFTTFEIVYPVLVILKCDSAVLSGFVLMFIACIVWLKLVSFAHTNHDIRK**LITS**
GKKVDNELTAAGIDNLQAPTLGSLTYFMMAPTLCYQPSYPRTPYVRKGWLVRQVILYLIFTGLQGFIIEQYINP
IVVNSQHPLMGGSYPYDVPDYAGSGLLNAVETVLKLSLPNVYLWLCMFYCLFHLWLNILAEILRFGDREFYKDW
WNAKTIDEYWRKWNMPVHKWIVRHIYFPCMRNGISKEVAVFISFFVSAVLHELCVAVPCHILKFWAFLGIMLQI
PLIILTSYLKNKFSDTMVGNMIFWFFFCIYGQPMCVLLYYHDVMNRTEKAK**GELRGHPFEGKPIPNPLLGLDST**
RTGHHHHHH

Xp:: ZmL-HA^{388}::V5 (*ON959587*)
MGGSHHHHHHGMASMTGGQQMGRDLYDDDDKVPRIQCGGIRRQKMADSEDAPPAVHRRPPRPARGAAAAQGFAA
ALRRRLRSGAAVAARASFAADSGDESGPGEPSSSRRRDNSGGASSAAGGRAGAGDFSAFTFRAAAPVHRKAKES
PLSSDAIFKQSHAGLFNLCIVVLVAVNSRLIIENLMKYGLLIRSGFWFNATSLRDWPLLMCCLSLPIFPLGAFA
VEKLAFNNLISDPATTCFHILFTTFEIVYPVLVILKCDSAVLSGFVLMFIACIVWLKLVSFAHTNHDIRK**LITS**
GKKVDNELTAAGIDNLQAPTLGSLTYFMMAPTLCYQPSYPRTPYVRKGWLVRQVILYLIFTGLQGFIIEQYINP
IVVNSQHPLMGGLLNAVETVLKLSLPNVYLWLCMFYCLFHLWLNILAEILRFGDREFYKDWGSYPYDVPDYAGS
WNAKTIDEYWRKWNMPVHKWIVRHIYFPCMRNGISKEVAVFISFFVSAVLHELCVAVPCHILKFWAFLGIMLQI
PLIILTSYLKNKFSDTMVGNMIFWFFFCIYGQPMCVLLYYHDVMNRTEKAK**GELRGHPFEGKPIPNPLLGLDST**
RTGHHHHHH

Xp::ZmL-HA^{473}::V5 (*ON959588*)

MGGSHHHHHHGMASMTGGQQMGRDLYDDDDKVPRIQCGGIRRQKMADSEDAPPAVHRRPPRPARGAAAAQGFAA
ALRRRLRSGAAVAARASFAADSGDESGPGEPSSSRRRDNSGGASSAAGGRAGAGDFSAFTFRAAAPVHRKAKES
PLSSDAIFKQSHAGLFNLCIVVLVAVNSRLIIENLMKYGLLIRSGFWFNATSLRDWPLLMCCLSLPIFPLGAFA
VEKLAFNNLISDPATTCFHILFTTFEIVYPVLVILKCDSAVLSGFVLMFIACIVWLKLVSFAHTNHDIRK**LITS
GKKVDNELTAAGIDNLQAPTLGSLTYFMMAPTLCYQPSYPRTP**YVRKGWLVRQVILYLIFTGLQGFIIEQYINP
IVVNSQHPLMGGLLNAVETVLKLSLPNVYLWLCMFYCLFHLWLNILAEILRFGDREFYKDWWNAKTIDEYWRKW
NMPVHKWIVRHIYFPCMRNGISKEVAVFISFFVSAVLHELCVAVPCHILKFWAFLGIMLQIPLIILTSYLKNGS
YPYDVPDYAGSKFSDTMVGNMIFWFFFCIYGQPMCVLLYYHDVMNRTEKAK**GELRGHPFEGKPIPNPLLGLDST
RTGHHHHHH**

Xp::ZmL::V5 (*ON959579*)

MGGSHHHHHHGMASMTGGQQMGRDLYDDDDKVPRIQCGGIRRQKMADSEDAPPAVHRRPPRPARGAAAAQGFAA
ALRRRLRSGAAVAARASFAADSGDESGPGEPSSSRRRDNSGGASSAAGGRAGAGDFSAFTFRAAAPVHRKAKES
PLSSDAIFKQSHAGLFNLCIVVLVAVNSRLIIENLMKYGLLIRSGFWFNATSLRDWPLLMCCLSLPIFPLGAFA
VEKLAFNNLISDPATTCFHILFTTFEIVYPVLVILKCDSAVLSGFVLMFIACIVWLKLVSFAHTNHDIRK**LITS
GKKVDNELTAAGIDNLQAPTLGSLTYFMMAPTLCYQPSYPRTP**YVRKGWLVRQVILYLIFTGLQGFIIEQYINP
IVVNSQHPLMGGLLNAVETVLKLSLPNVYLWLCMFYCLFHLWLNILAEILRFGDREFYKDWWNAKTIDEYWRKW
NMPVHKWIVRHIYFPCMRNGISKEVAVFISFFVSAVLHELCVAVPCHILKFWAFLGIMLQIPLIILTSYLKNKF
SDTMVGNMIFWFFFCIYGQPMCVLLYYHDVMNRTEKAK**GELRGHPFEGKPIPNPLLGLDSTRTGHHHHHH**

Bold green lettering MGGGS indicates the substitute sequence for N-terminus after truncation. Bold orange lettering AKGELRGHPFEGKPIPNPLLGLDSTRTGHHHHHH is the C-terminal V5-His tags used in the non N-terminally tagged full length, ΔN truncated and chimeric DGAT1s. Bold brown lettering GELRGHPFEGKPIPNPLLGLDSTRTGHHHHHH is the C-terminal V5-His tags used in the N-terminal Xp tagged and internally HA tagged ZmL DGAT1s. Bold blue lettering MGGSHHHHHHGMASMTGGQQMGRDLYDDDDKVP RIQCGGIRRQK shows the position and sequence of the N-terminal Xp tag. Underlined bold face LITSGKKVDNELTAAGIDNLQAPTLGSLTYFMMAPTLCY highlights the deduced position of the inter cytosolic loop in ZmL. Blue highlight GSYPYDVPDYAGS shows the position and sequence of the internal HA tag.
