# Supplementary material for: An alternative angiosperm DGAT1 topology and potential motifs in the N-terminus
Source: Front Plant Sci. 2022 Sep 16;13:951389. doi: 10.3389/fpls.2022.951389 (PMC9523541; doi:10.3389/fpls.2022.951389)
Supplement: Supplementary file 2 [file Table_2.pdf]

Supplmentary Table 2. Standard error (SE) values for means presented in Table 1.

	SE (±) of cell growth, fatty acid content and TAG content in *S. cerevisiae*						
**Construct**	**g/DW/L (8 h)**	**g/DW/L (24 h)**	**g/DW/L (48 h)**	**FA (% DW) (8 h)**	**FA (% DW) (24 h)**	**FA (% DW) (48 h)**	**TAG (% DW) (48 h)**
**Vc**	0.04	0.01	0.03	0.1	0.07	0.10	0
**At**	0.01	0.10	0.09	0.2	0.15	0.23	0.08
**Tm**	0.04	0.27	0.12	0.07	0.15	0.10	0.03
**OsS**	0.01	0.17	0.1	0.11	0.10	0.42	0.04
**ZmS**	0.05	0.23	0.26	0.18	0.26	0.18	0.04
**OsL**	0.03	0.21	0.18	0.09	0.03	0.28	0.04
**ZmL**	0.03	0.18	0.18	0.06	0.13	0.30	0.07
**ΔN At**	0.03	0.06	0.12	0.23	0.29	0.18	0.02
**ΔN Tm**	0.02	0.14	0.18	0.11	0.25	0.17	0.19
**ΔN OsS**	0.05	0.18	0.12	0.11	0.11	0.14	0.006
**ΔN ZmS**	0.06	0.23	0.25	0.15	0.25	0.23	0.04
**ΔN OsL**	0.02	0.09	0.23	0.03	0.32	0.42	0.12
**ΔN ZmL**	0.03	0.13	0.12	0.10	0.09	0.40	0.09
**At::ZmL**	0.02	0.08	0.16	0.11	0.06	0.08	0.005
**ZmL::At**	0.05	0.006	0.12	0.25	0.09	0.06	0.23
**Tm::ZmS**	0.006	0.06	0.22	0.38	0.15	0.16	0.1
**ZmS::Tm**	0.05	0.04	0.07	0.23	0.13	0.36	0.28
**Tm::ZmL**	0.01	0.09	0.22	0.33	0.22	0.27	40.1
**ZmL::Tm**	0.06	0.09	0.06	0.33	0.13	0.21	0.07
