# Supplementary material for: An alternative angiosperm DGAT1 topology and potential motifs in the N-terminus
Source: Front Plant Sci. 2022 Sep 16;13:951389. doi: 10.3389/fpls.2022.951389 (PMC9523541; doi:10.3389/fpls.2022.951389)
Supplement: Supplementary file 3 [file Table_3.pdf]

Supplementary Table 3. Predicted charge over a range of pH values of the cytoplasmically located variable N-terminal regions from a variety of plant DGAT1s.

	Predicted charge of cytoplasmic variable N-terminus											
	DGAT1											
**pH**	**ZmL**	**SbL**	**OsL**	**OsS**	**ZmS**	**Tm**	**Vg**	**SbS**	**Vv**	**Gm**	**Lj**	**At**	**Nt**
**5.0**	9.8	8.2	10.3	3.4	2.0	3.3	4.3	0.4	1.8	-0.4	-0.1	-1.4	0.2
**5.5**	8.5	6.6	8.4	2.3	0.6	1.6	2.0	-1.3	-0.2	-2.2	-2.2	-3.8	-2.6
**6.0**	7.7	5.8	7.1	1.7	-0.2	0.6	0.7	-2.2	-1.2	-3.2	-3.4	-4.8	-4.0
**6.5**	7.0	5.1	5.6	1.0	-1.0	-0.4	-0.4	-2.9	-1.9	-3.9	-4.4	-5.4	-4.9
**7.0**	6.4	4.4	4.1	0.4	-1.6	-1.3	-1.3	-3.6	-2.6	-4.6	-5.3	-5.8	-5.6
**7.5**	5.9	3.9	3.2	-0.1	-2.1	-2.0	-2.0	-4.1	-3.2	-5.1	-6.0	-6.1	-6.1
**8.0**	5.6	3.6	2.7	-0.4	-2.5	-2.4	-2.5	-4.5	-4.0	-5.5	-6.4	-6.5	-6.7
**8.5**	5.3	3.2	2.3	-0.8	-2.8	-2.8	-3.1	-4.8	-4.9	-5.9	-6.8	-6.8	-7.4
**9.0**	5.1	3.0	2.1	-1.0	-3.1	-3.2	-3.9	-5.1	-6.0	-6.3	-7.2	-7.1	-8.1

Amino acid sequences covering the N-terminus through to the conserved arginine residue immediately upstream of the predicted Acyl-CoA binding domain were submitted to Protein Calculator v3.4 (http://protcalc.sourceforge.net/) which calculated the charge from pH 5.0 to 9.0 (in 0.5 increments). Abbreviations: At = *A. thaliana*; Gm = *G. max*; Lj = *L. japonicas*; Nt = *N. tabacum*; OsS = *O. sativa* (Short); OsL *O. Sativa* (Long); SbS = *S. bicolor* (Short); SbL = *S. bicolor* (Long); Tm = *T. majus*; Vg = *V. galamensis*; Vv = *V. vinifera*; Zms = *Z. mays* (Short); ZmL = *Z. mays* (long).
