# Supplementary material for: An alternative angiosperm DGAT1 topology and potential motifs in the N-terminus
Source: Front Plant Sci. 2022 Sep 16;13:951389. doi: 10.3389/fpls.2022.951389 (PMC9523541; doi:10.3389/fpls.2022.951389)
Supplement: Supplementary file 4 [file Table_4.pdf]

Supplementary Table 4. Comparison of seed weight (mg), seed lipid content (% DW) in wild-type (WT) and vector control (VC) as well as individual lines of *Camelina sativa* seeds from plants that had been transformed with either a full length DGAT1, a ΔN DGAT1 or a chimeric DGAT1.

**Plant** *(n=8)*	**Seed Size (mg/seed)**			**Seed Lipid (%DW)**		
	**Average**	**SE**	***P-value***	**Average**	**SE**	***P-value***
**WT**	1.05	0.02		27.3	0.7	
**VC**	1.05	0.01	*0.7870*	26.5	0.6	*0.3988*
**Tm#1**	1.02	0.04	*0.1001*	24.9	0.4	*0.0335*
**Tm#2**	1.05	0.01	*0.7695*	29.5	0.6	*0.0364*
**Tm#5**	0.99	0.02	*0.0423*	28.8	1.2	*0.2811*
**Tm#9**	1.00	0.11	*0.0645*	25.3	0.8	*0.0711*
**ZmS#1**	1.01	0.02	*0.0731*	26.2	0.7	*0.2564*
**ZmS#15**	1.12	0.04	*2.26E-05*	29.2	1.3	*0.3033*
**ZmS#18**	1.01	0.01	*0.0260*	28.0	0.9	*0.5587*
**ZmL#1**	1.03	0.06	*0.6231*	26.0	2.0	*0.2873*
**ZmL#6**	0.84	0.02	*2.85E-07*	26.4	1.2	*0.5515*
**ZmL#9**	1.02	0.01	*0.0989*	30.1	0.5	*0.0037*
**NΔZmL#1**	1.13	0.01	*0.0051*	31.4	0.5	*0.0002*
**NΔZmL#2**	1.03	0.01	*0.2454*	31.6	0.5	*0.0002*
**Tm::ZmS#8**	0.94	0.01	*0.0006*	26.8	0.7	*0.6152*
**Tm::ZmS#9**	1.04	0.02	*0.6140*	32.5	0.3	*5.48E-06*
**Tm::ZmL#3**	1.15	0.03	*0.0031*	30.71	1.1	*0.0458*
**Tm::ZmL#4**	1.11	0.04	*0.0556*	33.6	1.18	*0.0014*
**Tm::ZmL#5**	1.03	0.01	*0.3464*	42.0	1.0	*2.13E-07*
**Tm::ZmL#13**	1.16	0.02	*0.0018*	37.2	0.2	*1.19E-09*
**ZmS::Tm#1**	1.34	0.04	*2.54E-07*	34.0	0.8	*0.0006*
**ZmS::Tm#3**	1.36	0.02	*4.42E-08*	39.3	1.2	*4.44E-07*
**ZmS::Tm#4**	1.51	0.01	*2.46E-13*	33.9	1.3	*0.0007*
**ZmL::Tm#22**	1.21	0.01	*1.73E-09*	32.9	0.6	*1.78E-05*
**ZmL::Tm#23**	1.04	0.02	*0.9492*	27.3	1.1	*0.7504*
