# Supplementary material for: An alternative angiosperm DGAT1 topology and potential motifs in the N-terminus
Source: Front Plant Sci. 2022 Sep 16;13:951389. doi: 10.3389/fpls.2022.951389 (PMC9523541; doi:10.3389/fpls.2022.951389)
Supplement: Supplementary file 5 [file Table_5.pdf]

Supplementary Table 5. Standard error (SE) values for means presented in Table 2.

	SE (±) of seed weight, % lipid content and mg lipid per seed in *C. sativa*					
**Plant**	**HOM Seed Size (mg/seed)**	**Null Seed Size (mg/seed)**	**HOM % Lipid/Seed (%DW)**	**Null % Lipid/Seed (%DW)**	**HOM Lipid/Seed (mg/seed)**	**Null Lipid/Seed (mg/seed)**
**WT**	0.02	0.01	1.2	0.5	0.014	0.005
**VC**	0.02	0.02	0.7	0.7	0.009	0.010
**Tm#2**	0.01	0.01	0.9	0.5	0.009	0.005
**Tm#5**	0.02	0.03	1.2	0.5	0.015	0.005
**ZmS#1**	0.02	0.01	0.5	0.7	0.010	0.009
**ZmS#18**	0.01	0.01	0.5	0.6	0.005	0.006
**ZmL#6**	0.01	0.01	0.5	0.2	0.005	0.006
**ZmL#9**	0.02	0.01	0.3	1.0	0.005	0.012
**NΔZmL#1**	0.01	0.01	0.6	0.8	0.008	0.010
**NΔZmL#2**	0.01	0.01	1.0	0.7	0.008	0.007
**Tm::ZmS#8**	0.01	0.03	0.7	0.6	0.009	0.008
**Tm::ZmS#9**	0.02	0.02	0.2	0.8	0.003	0.006
**ZmL::Tm#22**	0.02	0.01	1.2	0.8	0.021	0.007
**ZmL::Tm#23**	0.02	0.01	1.1	0.7	0.015	0.016
**Tm::ZmL#5**	0.01	0.01	1.3	0.6	0.024	0.004
**Tm::ZmL#13**	0.02	0.01	1.2	0.5	0.014	0.005
**ZmS::Tm#3**	0.02	0.02	0.7	0.7	0.009	0.010
**ZmS::Tm#4**	0.01	0.01	0.9	0.5	0.009	0.005
