# Supplementary material for: An alternative angiosperm DGAT1 topology and potential motifs in the N-terminus
Source: Front Plant Sci. 2022 Sep 16;13:951389. doi: 10.3389/fpls.2022.951389 (PMC9523541; doi:10.3389/fpls.2022.951389)
Supplement: Supplementary file 6 [file Table_6.pdf]

Supplementary Table 6. Standard error (SE) values for means presented in Table 3.

	SE (±) of FA species (as a % of total FA) in *C. sativa* seeds												
**Plant**	**C16:0**	**C18:0**	**C18:1**	**C18:2**	**C18:3**	**C20:1**	**C20:2**	**C20:3**	**C22:0**	**C22:1**	**C22:3**	**C24:0**	**C24:1**
**WT**	0.072	0.082	0.30	0.39	0.55	0.17	0.033	0.034	0.025	0.18	0.041	0.017	0.048
**Tm**	0.044	0.022	0.037	0.10	0.065	0.040	0.020	0.017	0.018	0.029	0.030	0.027	0.018
**ZmS**	0.034	0.026	0.16	0.011	0.24	0.14	0.037	0.057	0.045	0.13	0.026	0.025	0.006
**ZmL**	0.075	0.059	0.34	0.63	0.71	0.090	0.057	0.057	0.029	0.19	0.019	0.020	0.044
**ΔN ZmL**	0.035	0.006	0.18	0.028	0.11	0.054	0.008	0.008	0.033	0.11	0.026	0.006	0.01
**Tm::ZmS**	0.100	0.076	0.67	0.63	0.80	0.44	0.072	0.061	0.045	0.29	0.074	0.025	0.094
**Tm::ZmL**	0.100	0.081	0.30	0.23	0.62	0.34	0.14	0.026	0.062	0.33	0.047	0.078	0.10
**ZmS::Tm**	0.044	0.014	0.22	0.46	0.48	0.13	0.026	0.050	0.013	0.15	0.041	0.014	0.037
**ZmL::Tm**	0.024	0.020	0.22	0.15	0.38	0.061	0.019	0.019	0.007	0.43	0.022	0.007	0.007
