# Supplementary material for: An alternative angiosperm DGAT1 topology and potential motifs in the N-terminus
Source: Front Plant Sci. 2022 Sep 16;13:951389. doi: 10.3389/fpls.2022.951389 (PMC9523541; doi:10.3389/fpls.2022.951389)
Supplement: Supplementary file 7 [file Image_1.pdf]

Supplementary Figure 1. Phylogenetic grouping of exon one peptide sequences from plant DGAT1s.

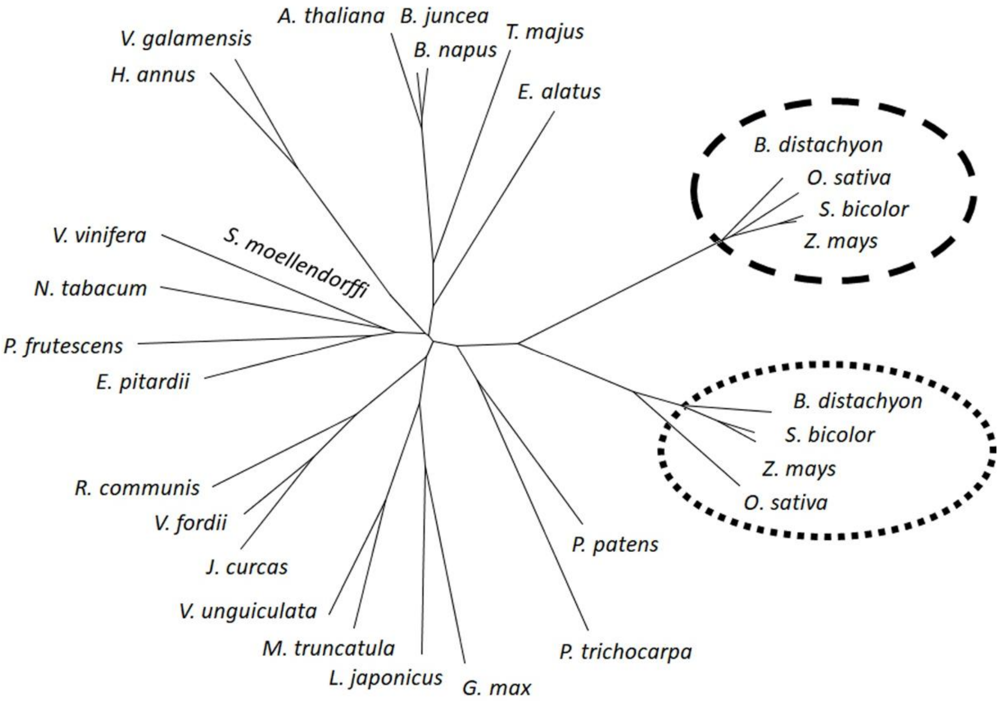

Accession numbers in addition to Figure 1 as follows: *B. distachyon* (Short), XP_003560655; *B. distachyon* (Long), XP_003568769; *E. pitardii*, ACO55635; *H. annus*, LC384987; *P. patens*, XP_024385355; *P. trichocarpa*, XP_006371996; *S. moellendorffi*, XP_002964165; *V. unguiculata*, XM_028049589 were aligned. From this it can be seen that the plant DGAT1 exon one peptide sequences generally grouped into clades corresponding to their taxonomic family. The Poaceae DGAT1s formed two discreet clades, one containing all the shortest sequences (highlighted by the dotted circle) and the other containing the longer sequences (highlighted by the dashed circle). Each grass species was found to have a representative in both clades.
