# Supplementary material for: An alternative angiosperm DGAT1 topology and potential motifs in the N-terminus
Source: Front Plant Sci. 2022 Sep 16;13:951389. doi: 10.3389/fpls.2022.951389 (PMC9523541; doi:10.3389/fpls.2022.951389)
Supplement: Supplementary file 8 [file Image_2.pdf]

Supplementary Figure 2. Nucleotide sequencing analysis of the plant DGAT1s.

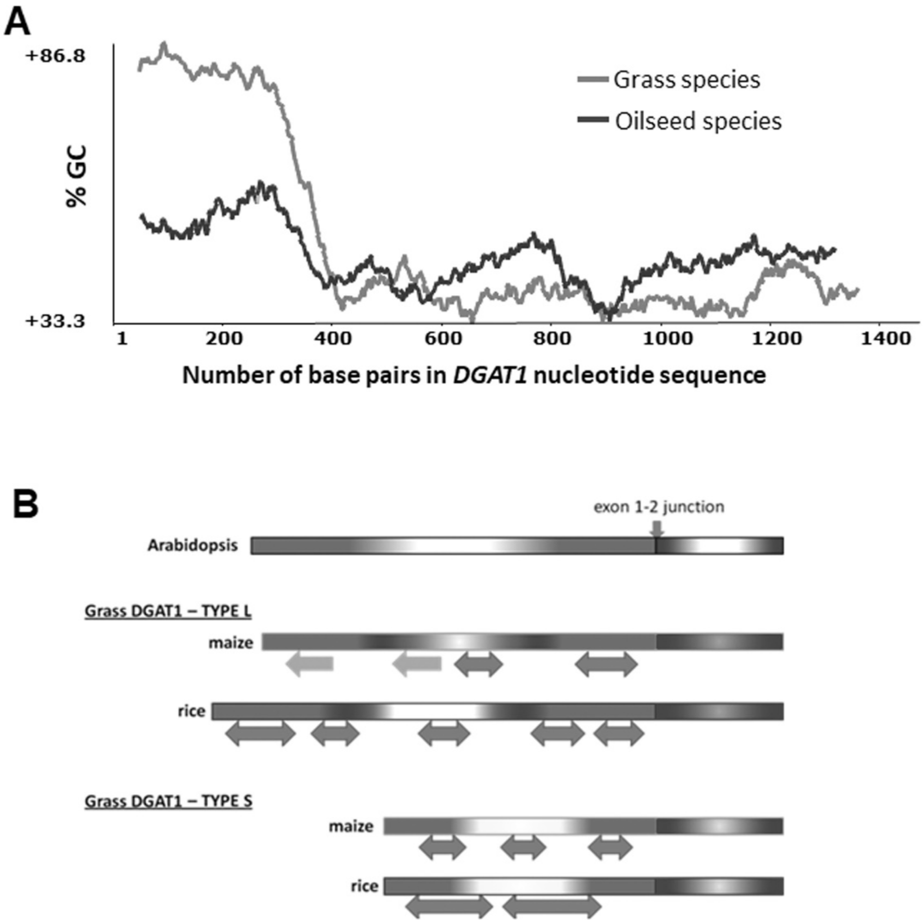

A) Sequencing analysis of grass DGAT1 showed a very high GC content in the region of exon1, compared to the oilseed crop DGAT1s. **B)** DGAT1 exon 1 nucleic acid sequences from the Gramineae family appeared to contain a number of palindromic repeats with uneven ends. Long exon 1 Gramineae sequences are referred to as Type L; while short exon 1 Gramineae sequences are referred to as Type S. The positions of the palindrome sequences are shown schematically for the Type S and for the Type L sequences; directionality indicates direct and inverse repeats.
