# Supplementary material for: An alternative angiosperm DGAT1 topology and potential motifs in the N-terminus
Source: Front Plant Sci. 2022 Sep 16;13:951389. doi: 10.3389/fpls.2022.951389 (PMC9523541; doi:10.3389/fpls.2022.951389)
Supplement: Supplementary file 9 [file Image_3.pdf]

Supplementary Figure 3. Immunoblot analysis of recombinant DGAT1s in the total cell protein extracts.

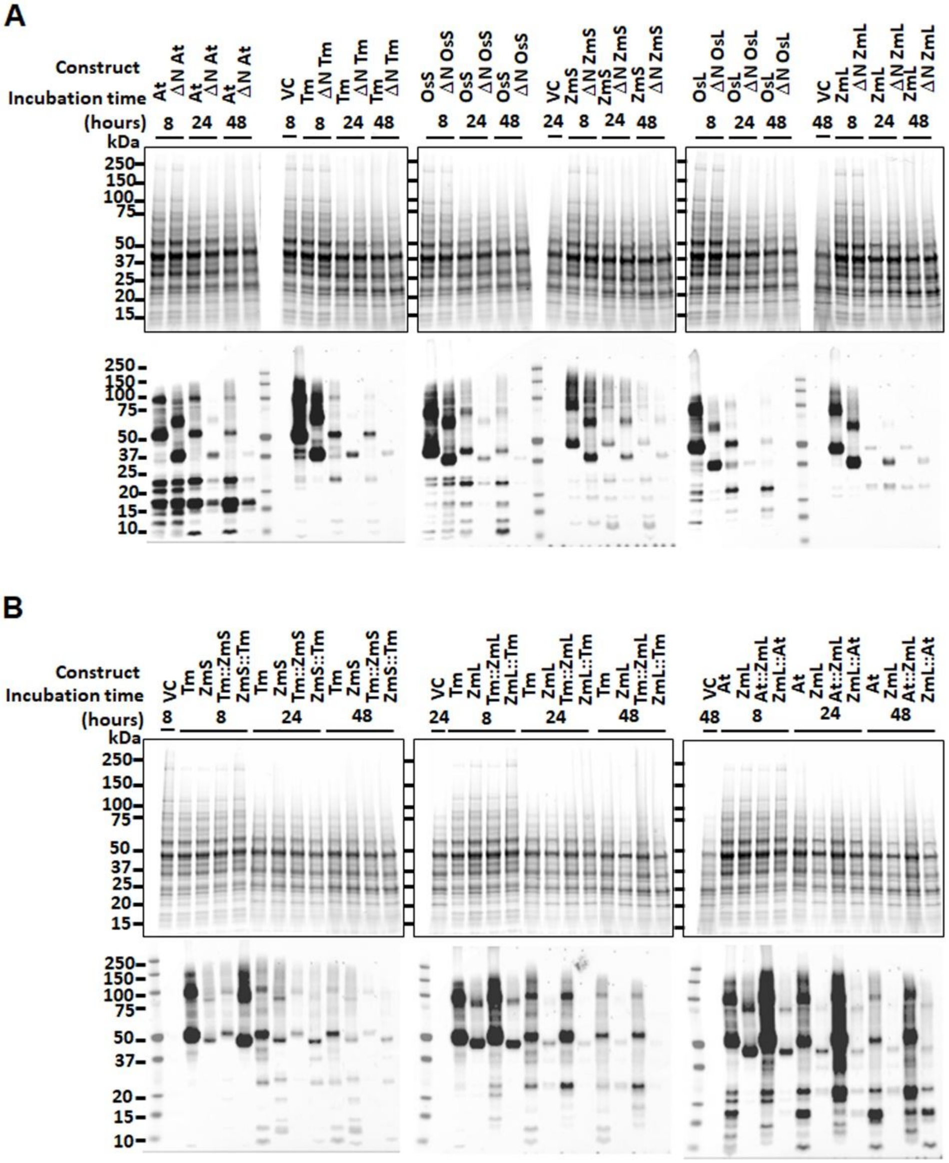

Immunoblot analysis of recombinant DGAT1s in total cell proteins, extracted from 8, 24 and 48 h cultures (left, centre, right respectively). Blots were probed with anti V5 antibodies. Gel loadings are shown by the in-gel stain-free image at the top of each immunoblot. Loading was based on equal quantities cell dry weight extract. **A)** Extracts from cells expressing full length At, Tm, OsS, ZmS, OsL, and ZmL and their N-terminally truncated forms (ΔN). **B)** Extracts from cells expressing full length At, Tm, ZmS, and ZmL and the chimeras Tm::ZmS, ZmS::Tm, Tm::ZmL, ZmL::Tm, At::ZmL, and ZmL::At.
