# Supplementary material for: An alternative angiosperm DGAT1 topology and potential motifs in the N-terminus
Source: Front Plant Sci. 2022 Sep 16;13:951389. doi: 10.3389/fpls.2022.951389 (PMC9523541; doi:10.3389/fpls.2022.951389)

Supplementary Figure 4. Microsomal protein quantities recovered from yeast cultures expressing either full length, ΔN DGAT1s or a chimeric DGAT in the pYES2.1/V5-His-TOPO yeast expression vector (Life Technologies, K4150-01). At least two independent culture incubations were performed.

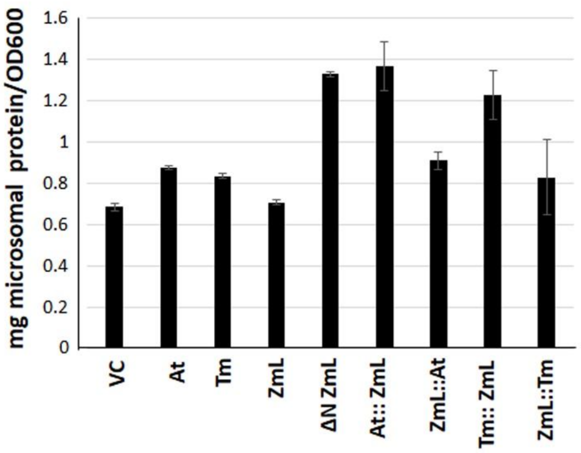

Supplement: Supplementary file 10 [file Image_4.pdf]
