# Supplementary material for: An alternative angiosperm DGAT1 topology and potential motifs in the N-terminus
Source: Front Plant Sci. 2022 Sep 16;13:951389. doi: 10.3389/fpls.2022.951389 (PMC9523541; doi:10.3389/fpls.2022.951389)
Supplement: Supplementary file 11 [file Image_5.pdf]

Supplementary Figure 5. Recombinant DGAT1 in the fat pad from *Saccharomyces cerevisiae* and lipid droplets from *Camelina sativa*.

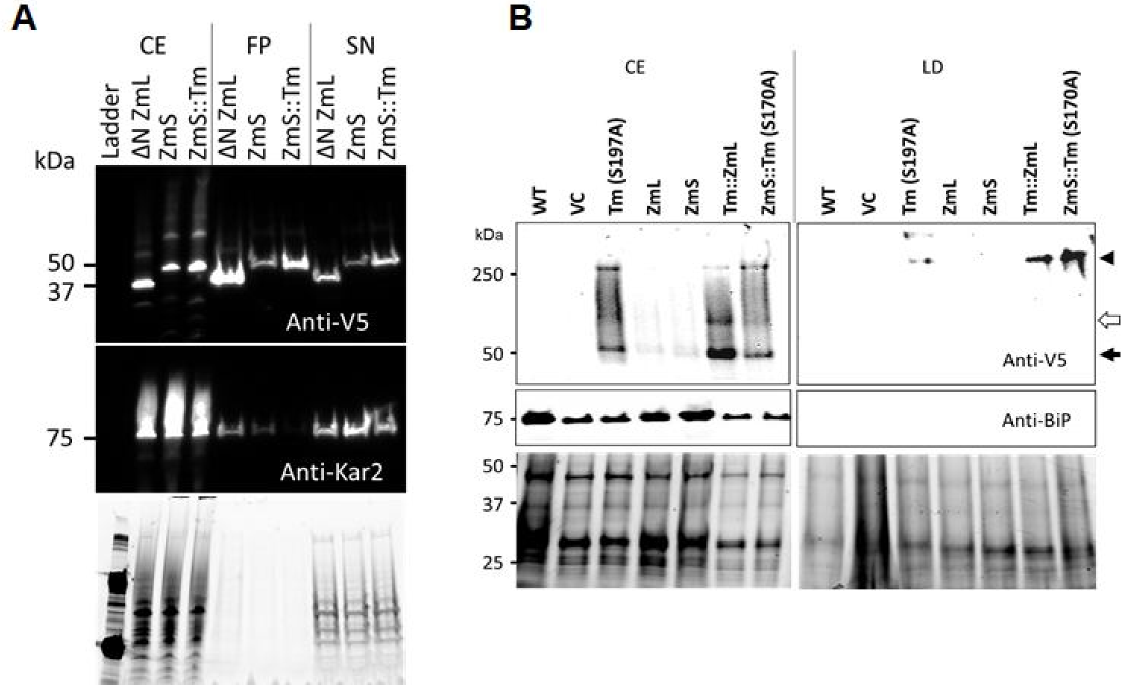

A) *S. cerevisiae* cells expressing ΔN ZmL; ZmS or ZmS::Tm were extracted for total protein, the extract was subjected to relatively low speed (20, 000g) centrifugation to isolate the fat pad (FP). Fractions were subsequently analysed for the presence of recombinant protein; including: the crude extract (CE), fat pad (FP), and underlying supernatant (SN). The fractions were also examined for the ER marker protein Kar2. Given the level of total protein loaded (bottom panel) the FP shows a very high level of enrichment of the DGAT seen by strong V5 signal in comparison to the signals seen in the CE and SN. In comparison, Kar2 signal was barely detected in the FP indicating the majority of DGAT found in the FP fraction is not associated with microsomal contamination.

B) *C. sativa* seeds expressing Tm, ZmL, ZmS, Tm::ZmL and ZmS::Tm were extracted for total protein, the extract was subjected to low speed (10, 000g) centrifugation to isolate the lipid droplets (LDs). The crude extract (CE) and the LDs were subsequently analysed for the presence of the recombinant protein and ER marker protein (BiP). The levels of ZmL and ZmS detected were considerably lower than Tm, Tm::ZmL and ZmS::Tm. In the CE the recombinant DGAT1s electrophoresed predominantly as monomers (solid arrow), although dimers (open arrow) and large oligomers (solid arrowhead) were present in some of the extracts. In the LD recombinant Tm::ZmL, ZmS::Tm and Tm were present (only as large oligomers); in contrast BiP was not detected indicating that the DGAT1 was not from microsomal contamination. The levels of DGAT1 detected in the LDs was positively correlated with the levels of the recombinant DGAT in the seeds and likely explains the faint ZmS and ZmL signals in the CE and lack of signal in the LDs.
