# Supplementary material for: An alternative angiosperm DGAT1 topology and potential motifs in the N-terminus
Source: Front Plant Sci. 2022 Sep 16;13:951389. doi: 10.3389/fpls.2022.951389 (PMC9523541; doi:10.3389/fpls.2022.951389)
Supplement: Supplementary file 12 [file Image_6.pdf]

Supplementary Figure 6. Immunoblot analysis of the DGAT1 C-terminal V5 tag in yeast microsomes, before and after trypsin protease digestion.

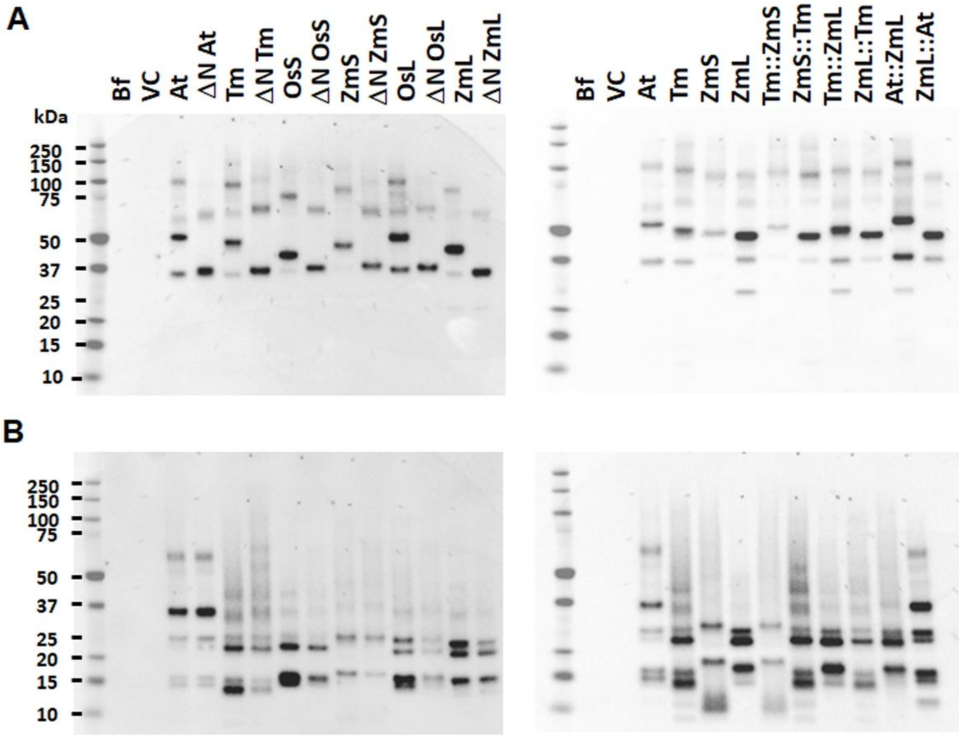

The majority of undigested total protein extracts (**A**, upper panels) from the microsomal preparations had two immunoreactive bands; the main band represents the appropriate DGAT1 in the monomeric form while the fainter larger bands are presumably homodimers. Following incubation with trypsin (**B**, lower panels) immunoreactive bands were detected in all DGAT1 lanes indicating the C-terminus was protected from digestion and as such is located in the lumen of the ER. In the majority of cases two predominant immunoreactive bands appeared after digestion, one at approximately 13-17 kDa and the other at approximately 25 kDa; these represent the luminally located C-terminal monomer and its dimer respectively. The change in ratio between the monomer and dimer from the top panels to the bottom panels may be a reflection of the increased overall hydrophobicity of the remaining C-terminal fragment compared to the full-length protein.
