# Supplementary material for: An alternative angiosperm DGAT1 topology and potential motifs in the N-terminus
Source: Front Plant Sci. 2022 Sep 16;13:951389. doi: 10.3389/fpls.2022.951389 (PMC9523541; doi:10.3389/fpls.2022.951389)

Supplementary Figure 7. The overall three-dimensional predicted structure of the maize ZmL DGAT1 (XP_008648150, AlphaFold (https://alphafold.ebi.ac.uk) resembles the nine α-helix structure of hydrophobic domains (orange circles) observed with exposed N and C-termini in opposite direction.

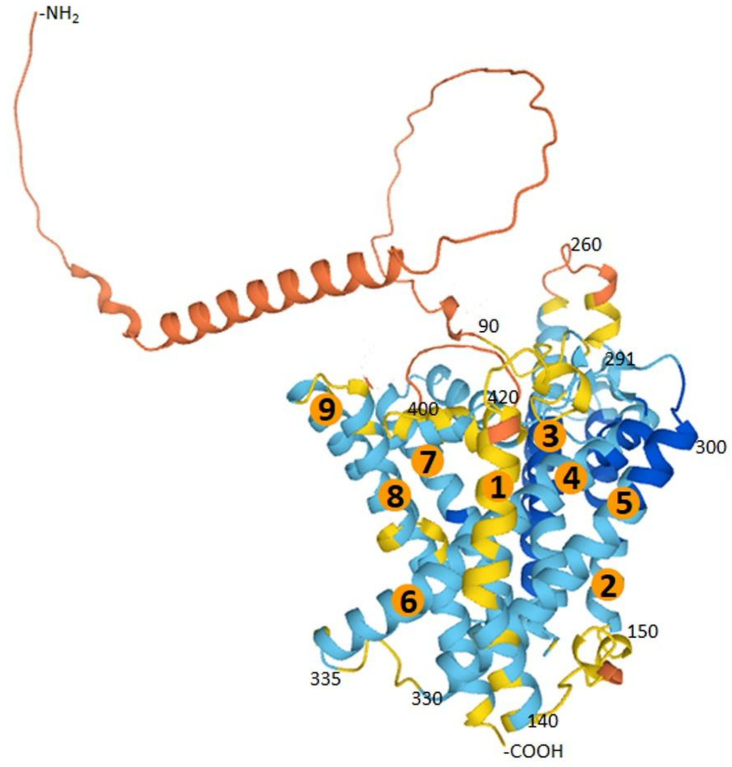

Supplement: Supplementary file 13 [file Image_7.pdf]
